# Characteristics, treatment regimens, and outcomes of patients with true extramedullary multiple myeloma: a real-world monocentric analysis

**DOI:** 10.1007/s00277-026-07118-6

**Published:** 2026-06-16

**Authors:** Marie Harzer, Nils Gross-Fengels, Fede Melzer, Isabel Molwitz, Ricardo Kosch, Maximilian Al-Bazaz, Jule Artzenroth, Leon Cords, Leandra Bartke, Abdulaziz Kamili, Winfried Alsdorf, Carsten Bokemeyer, Katja Weisel, Lisa B. Leypoldt, Christoph Schaefers

**Affiliations:** 1 Department of Oncology, Hematology and Bone Marrow Transplantation With Division of Pneumology, University Medical Center Hamburg-Eppendorf, Martinistraße 52, 20246 Hamburg, Germany; 2 Department of Diagnostic and Interventional Radiology and Nuclear Medicine, University Medical Center Hamburg-Eppendorf, Hamburg, Germany; 3 Mildred-Scheel-Nachwuchszentrum, Universitätsklinikum Hamburg-Eppendorf, Hamburg, Germany

**Keywords:** Multiple myeloma, Extramedullary disease, Plasma cell leukemia

## Abstract

Despite substantial therapeutic advances in multiple myeloma (MM), extramedullary disease (EMD) remains an aggressive subtype associated with poor prognosis, for which consensus on management is lacking and dedicated clinical trials are scarce. We conducted a retrospective single-center analysis of 86 patients with radiologically confirmed EMD, defined as soft-tissue involvement without bone contiguity (data cut-off September 30, 2024). The cohort included patients with *de novo* EMD (*n* = 19, 22%) and *secondary* EMD at relapse of MM (*n* = 67, 78%). We assessed clinical characteristics, survival outcomes, and treatment strategies. Treatment was highly heterogeneous, comprising > 50 distinct regimens. Median overall survival (mOS) from initial MM diagnosis was 55 months. mOS from EMD occurrence was 28 months for *de novo* and 21 months for *secondary* EMD. Survival varied by anatomical site: central nervous system (CNS), pulmonary, and retroperitoneal involvement showed a trend toward inferior survival, whereas lymph node involvement was associated with significantly longer survival in exploratory analyses (*p* = 0.011). Based on these findings, we exploratorily categorized anatomical sites into a three-tiered risk grouping, which revealed a stepwise gradient that did not reach statistical significance (*p* = 0.054). High-risk cytogenetic features were present in 50% of patients with *de novo* EMD and in 43% with *secondary EMD*. Novel agents, including CAR T-cell therapy and bispecific antibodies, were used predominantly in later lines, with responses in secondary EMD that were often transient. This real-world analysis confirms the high-risk nature of EMD, especially for patients with CNS, pulmonary, or retroperitoneal involvement. Given the observed treatment heterogeneity, prospective registries are urgently needed to define optimal therapeutic sequencing and address the high unmet therapeutic need in EMD patients.

**Supplementary Information:**

The online version contains supplementary material available at 10.1007/s00277-026-07118-6.

## Introduction

Multiple Myeloma (MM) is a malignant, biologically heterogeneous plasma cell neoplasm characterized by the clonal expansion of plasma cells (PCs) within the bone marrow (BM) microenvironment [1–4]. The expansion of a PC subclone outside the BM, driven by the loss of BM homing mechanisms, represents an aggressive MM subtype, termed extramedullary disease (EMD), and is associated with a poor prognosis [5–8].

EMD has been inconsistently defined in the literature, leading to discrepancies in its classification [3, 4, 8]. Some studies include soft-tissue involvement arising from adjacent bone lesions through cortical bone erosion (paraskeletal disease, PSD) within the spectrum of EMD**.** However, a more recent definition proposed by Rosiñol et al. restricts EMD to non-bone-related extramedullary manifestations, characterized by PC infiltration of soft tissues, visceral organs, or the central nervous system (CNS), without contiguity to bone lesions (true EMD) [3, 4, 8–12]. A clear distinction between true EMD and PSD is essential, as high-risk molecular features, enhanced proliferative activity, and resistance to therapy characterize true EMD. In contrast, PSD generally lacks these unfavorable features and shows a prognosis comparable to BM-only MM [3, 13–17]. This distinction is supported by a recent multicenter retrospective study evaluating outcomes of patients treated with standard-of-care (SOC) chimeric antigen receptor T-cell (CAR T) therapies in relapsed/refractory MM (RRMM), in which patients with PSD demonstrated survival outcomes similar to those observed in BM-only disease [17].

Previous studies have reported the incidence of EMD ranging from 0.5% to 5.2% in newly diagnosed MM (*de novo* EMD) and from 1.6% to 30% at disease relapse (*secondary* EMD). This wide variation in reported incidence rates may be attributed to differing definitions of EMD, as discussed above, as well as heterogeneity in diagnostic approaches across studies. In addition, an increasing incidence of EMD has been observed over time, a trend likely attributable to improved survival in the era of modern therapies, allowing patients to progress to disease phases that historically occurred less frequently [2, 3, 6–8, 10, 15, 17, 18]. Whereas patients with newly diagnosed MM have a projected median overall survival (mOS) of more than 10 years, the presence of EMD is consistently associated with significantly inferior outcomes, including shorter OS and progression-free survival (PFS) [3, 17, 19, 20]. A retrospective analysis by Zanwar et al. reported an mOS of 3.6 years for *de novo* EMD, whereas *secondary* EMD demonstrated an even worse mOS of only 0.7 years [14]. In line with these findings, a recent post hoc analysis of the pooled LocoMMotion and MoMMent studies confirmed inferior outcomes in triple-class-exposed (TCE) RRMM patients with EMD compared with those without EMD. Under currently available SOC therapy, patients with EMD showed lower overall response rates (ORR) (24.1% vs. 33.3%) and shorter mOS (7.16 months vs. 15.51 months) [21].

EMD is considered an independent high-risk feature, with frequent TP53 abnormalities and recurrent oncogenic driver mutations, especially RAS/BRAF mutations, which are reported to play a substantial role in the development of extramedullary spread [20]. In addition, studies have demonstrated an enrichment of genomically defined high-risk MM, including del17p, gain or amplification of 1q, and translocation t(4;14). Furthermore, EMD has been associated with immature or plasmablastic morphology, elevated lactate dehydrogenase (LDH) levels, and Revised International Staging System (R-ISS) stage III. CNS involvement, in particular, is linked to the most unfavorable prognosis [3, 4, 6, 8, 14, 18, 21–25].

Conventional chemotherapies and novel agents have shown limited effectiveness in patients with EMD [3, 17, 22, 26]. Despite significant advances in the treatment of MM with novel immunotherapeutics, such as chimeric antigen receptor T-cell (CAR T-cell) therapies and T-cell-redirecting bispecific antibodies (bsAbs), these approaches have not yet translated into durable long-term outcomes in patients with EMD [3, 14, 22, 27–29]. A retrospective analysis of anti-BCMA CAR T-cell clinical trials in MM reported ORRs ranging from 57 to 100%. However, these responses were often transient, as both PFS and OS remained shorter in patients with EMD compared with those without [22]. Most recently, the phase 2 RedirecTT-1 trial prospectively evaluated dual-antigen targeting with talquetamab plus teclistamab in patients with true EMD, reporting an ORR of 79% [30].

To date, no consensus on optimal therapeutic management for patients with EMD exists, and prospective randomized clinical trials specifically addressing this population are scarce, resulting in limited treatment efficacy [3, 14, 17, 21]. The scarcity of structured clinical guidance is further aggravated by an increase in EMD incidence [17, 31].

Given these considerations, we conducted a retrospective real-world analysis to characterize routine clinical management and to identify potential risk factors in patients with EMD treated at our tertiary center, with the aim of informing treatment decisions for this high-risk population. We report our experience with a cohort of MM patients with radiologically defined true EMD, with or without concurrent plasma cell leukemia (PCL). We analyzed demographic and baseline characteristics, common anatomical sites of EMD, treatment strategies and responses, and survival outcomes.

## Methods

### Study cohort

MM patients with radiologically confirmed EMD, with or without cytomorphologically confirmed PCL in peripheral blood smears, were retrospectively identified using electronic medical records (EMR) from a single tertiary academic center (University Medical Center Hamburg-Eppendorf). The initial diagnosis of MM in the included cohort ranged from December 1994 to April 2024, whereas EMD was diagnosed between April 2010 and September 2024. The data cut-off for this analysis was September 30, 2024.

EMD was defined on the basis of existing imaging reports from clinical routine (MRI, CT, or ^1^⁸F-FDG PET/CT) assessed by a radiologist with 2 years of experience and verified by a radiologist with 7 years of experience. When available, histopathological confirmation was obtained by biopsy or cerebrospinal fluid analysis in cases of CNS involvement. *De novo* EMD was defined as the presence of EMD identified within one month following the initial diagnosis of MM, whereas later occurrence was defined as *secondary* EMD.

PCL was defined according to the International Myeloma Working Group (IMWG) criteria as ≥ 5% plasma cells detected in the peripheral blood smear [32].

### Patient identification and screening process

The EMR database was systematically searched using the keyword “extramedullär” in radiologic reports between January 1, 2010, and September 30, 2024. This search yielded 1467 radiologic reports. All reports were manually reviewed to identify potential cases of extramedullary involvement in MM.

A total of 1323 reports were excluded in the initial review, including reports describing extramedullary hematopoiesis (e.g., myelofibrosis, thalassemia), non-myeloma malignancies (e.g., lymphoma, meningioma), benign lesions, or reports explicitly stating no evidence of EMD in MM, as well as reports explicitly describing PSD, defined as extraosseous plasma cell masses with contiguity to adjacent bone.

**Fig. 1:**
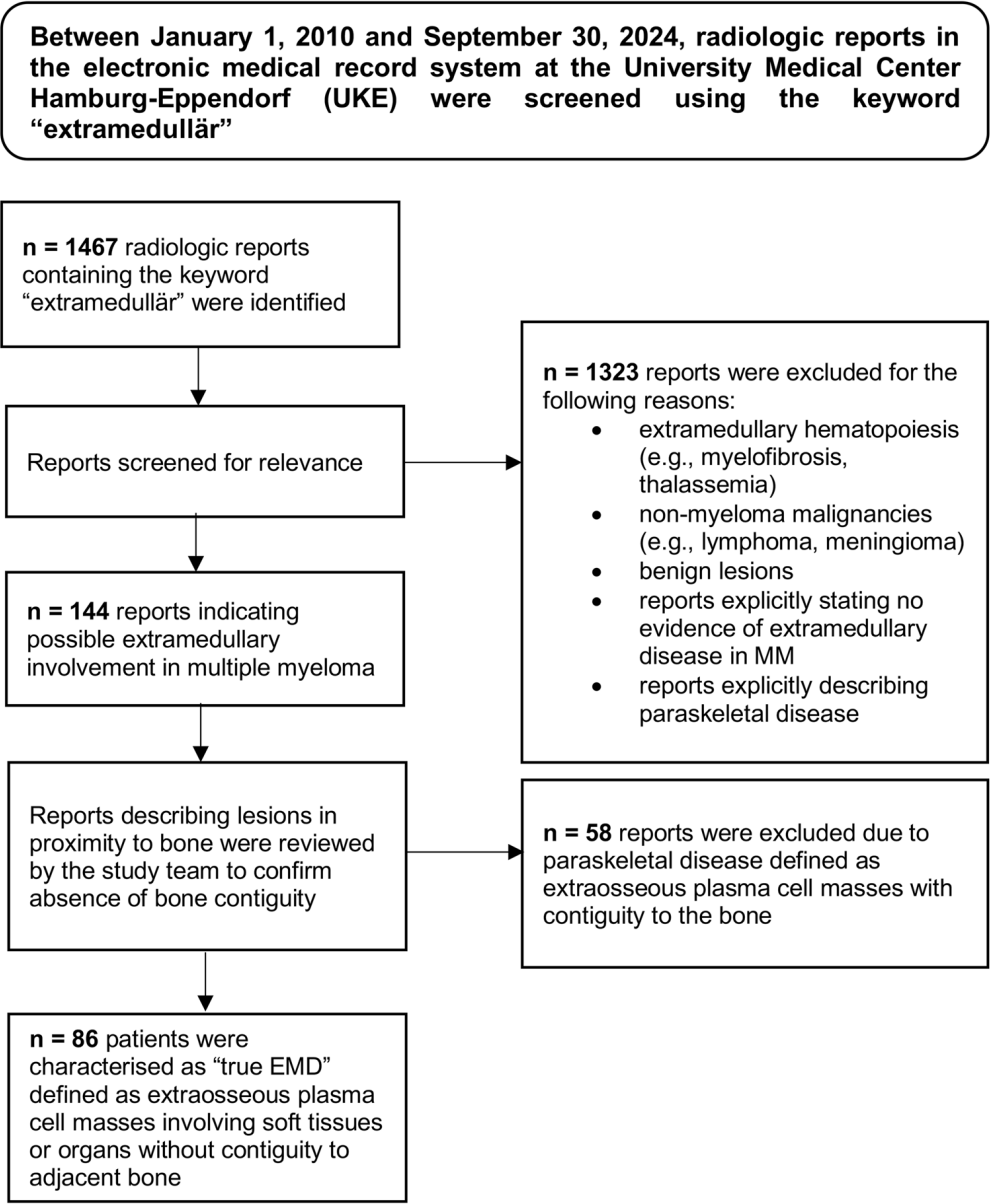
Identification and selection process of patients with true extramedullary disease (EMD). Flow diagram illustrating the screening and selection of radiologic reports in the electronic medical record database at the University Medical Center Hamburg-Eppendorf (UKE) between January 1, 2010 and September 30, 2024 using the keyword “extramedullär”

In a second screening step, radiologic reports describing EMD lesions in proximity to bone were reviewed by the study team to confirm the absence of bone contiguity and to ensure correct classification as true EMD rather than PSD. After exclusion of additional 58 cases classified as PSD, 86 patients with radiologically confirmed extraosseous plasma cell masses involving soft tissues or organs without contiguity to adjacent bone (true EMD) were included in the final study cohort. The identification and selection process are illustrated in Fig. 1.

### Data assessment

Treatment response was assessed according to the IMWG criteria [31]. Patients with oligo- or asecretory disease were excluded from evaluation of serologic response. Whenever feasible, serologic and radiologic response were assessed and categorized according to IMWG [31]. When both serologic and radiologic responses were available, the overall response was assigned based on the less favorable category according to IMWG [31]. In cases where radiological follow-up of EMD was not available, response assessment was based solely on serologic parameters (indicated by the prefix ‘se’ preceding the response category). All response data are reported at the line-of-therapy level, as individual patients may have received the same drug class across multiple lines of therapy. Cytogenetic risk stratification was performed according to the R-ISS staging system, with high-risk cytogenetic features defined as del(17p) and/or t(4;14) and/or t(14;16) [33].

Data from a subset of patients with EMD treated with CAR T-cell therapy or bsAbs had previously been reported by Steinhardt et al. in a multicenter retrospective analysis [34].

### Statistical analysis

Data concerning patients’ characteristics, treatment regimens, and outcome variables were analyzed using descriptive statistics. OS was defined as the time from initial diagnosis of MM to death from any cause. OS from EMD was defined as the time from initial diagnosis of EMD to death from any cause (OS-EMD). Relapse and lines of treatment were defined according to IMWG [31].

The OS was the primary endpoint and analyzed using Kaplan–Meier estimates. Median follow-up time was estimated using the reverse Kaplan–Meier method. PFS was defined as the time from initiation of each line of therapy to disease progression or death from any cause, whichever occurred first. PFS values of less than one month were coded as 0.5 months. Patients without an event were censored at the date of last follow-up. Patients receiving best supportive care (BSC) only were excluded from PFS calculation.

Univariable and multivariable Cox proportional hazards models were used to evaluate associations with overall survival. OS was analyzed from initial MM diagnosis and from first EMD diagnosis. For OS from MM diagnosis, candidate covariates included sex, age at MM diagnosis, ECOG performance status, ISS stage, R-ISS stage, cytogenetic risk, bone marrow plasma cell infiltration, and primary refractoriness. A full multivariable Cox model including all candidate covariates was fitted. In addition, a reduced multivariable model was evaluated including age, ECOG performance status, ISS stage, R-ISS stage, and primary refractoriness, selected based on univariable association, clinical relevance, and the limited sample size. For OS from EMD diagnosis, candidate covariates included sex, EMD at initial diagnosis, age at EMD, prior lines/exposure/refractoriness/BCMA therapy, and EMD organ sites. A full multivariable Cox model including all prespecified candidate covariates was fitted. In addition, a reduced multivariable model including lymph node involvement was evaluated based on univariable associations and clinical relevance. Missing observations were excluded on a model-wise complete-case basis by the Cox model fitting functions. Formal assessment of the proportional-hazards assumption was performed using scaled Schoenfeld residuals (cox.zph). No significant violations were observed in any model.

All site-specific survival analyses were considered exploratory. Due to the exploratory nature of these analyses, no adjustment for multiplicity was performed.

All statistical analyses were performed using R (v4.4.2) with the gtsummary (v2.0.4), survival (v3.8–3), finalfit (v1.0.8), and survminer (v0.5.0) packages and GraphPad Prism (v 10.3.1). Plots were generated with ggplot2 (v3.5.1) and GraphPad Prism (v 10.3.1).

## Results

This analysis includes 86 patients from our database, all presenting with radiologically confirmed true EMD not adjacent to bone. EMD was observed in 19 patients (22%) at initial diagnosis (*de novo* EMD). The remaining 67 patients (78%) developed EMD at relapse (*secondary* EMD), with a median time from initial diagnosis to EMD occurrence of 35 months (range, 3–287 months). Of these, EMD occurred at first relapse in 19 patients (28%), at second relapse in 10 patients (15%), and in later relapses in 38 patients (57%). Histological confirmation of EMD was obtained by biopsy in 15 patients (79%) with de novo EMD and in 38 patients (57%) with *secondary* EMD (total 62%). The median age at initial diagnosis of MM was 57.0 years and at first occurrence of EMD was 62.0 years. The study population was predominantly male (73%). Among evaluable patients (*n* = 58), 87% had an ECOG score of 0 or 1. At the time of initial diagnosis, most patients (77%) presented with International Staging System (ISS) stage II or III disease. Cytogenetic data, as determined by FISH analysis, were available for 76 of 86 patients (88.4%) at the time of initial MM diagnosis. High-risk cytogenetic features were observed in 50% of patients with *de novo* EMD and in 43% with *secondary* EMD, with del(17p) being the most frequent alteration (*de novo* EMD 44%, *secondary* EMD 31%), followed by t(4;14) (12%, 10%), and t(14;16) (0%, 5%). For de novo EMD, the median bone marrow infiltration was 35.0% (range, 0–100%; data available for 16/19 patients). For patients with secondary EMD, median BM infiltration at initial diagnosis was 63% (range, 8–100%; data available for 55/67 patients) and 35% (range, 0–100%; data available for 33/67 patients) at the time of EMD occurrence. Non-secretory disease was rare, occurring in three patients (3.5%). A total of 74 patients (86%) presented with EMD without concurrent PCL, whereas 12 patients (14%) had concurrent PCL. Five were initially diagnosed with PCL and subsequently developed EMD at relapse.

Among the 67 patients who developed EMD at relapse, this subgroup was heavily pretreated, with a median of 3 prior lines of therapy (range, 1–11). A substantial proportion of patients were triple-class exposed (24 patients, 36%), and 11 patients (16%) were penta-drug exposed. In total, 16 patients (24%) were triple-class refractory, and 9 (13%) were penta-drug refractory.

Regarding treatment prior to EMD, 56 patients (84%) had received high-dose melphalan followed by autologous stem cell transplantation (HDT/ASCT), predominantly as part of first-line therapy (*n* = 48). 14 patients underwent HDT/ASCT at relapse, including single or repeat courses. Seventeen patients had previously undergone allogeneic stem cell transplantation (alloSCT). 4 patients (6%) had already received novel therapies, including CAR T-cell therapy (*n* = 2), bsAb (*n* = 1), or antibody–drug conjugates (*n* = 2), with one patient receiving both CAR T and bsAb. Comprehensive baseline characteristics are summarized in Table 1.

**Table 1:** Baseline characteristics of patients in the study cohort with EMD and stratified by de novo and secondary EMD

	Whole cohort (*N* = 86)	*De novo* EMD (*N* = 19)	*Secondary* EMD (*N* = 67)
Sex			
male	63 (73%)	13 (68%)	50 (75%)
female	23 (27%)	6 (32%)	17 (25%)
Age at initial diagnosis of MM (years)	57.0 (50.0–65.0) [37.0–80.0]	57.0 (49.0–68.0) [42.0–80.0]	57.0 (50.0–65.0) [37.0–78.0]
ECOG at initial diagnosis of MM^1^			
0	20 (34%)	4 (27%)	16 (37%)
1	31 (53%)	11 (73%)	20 (47%)
2	6 (10%)	0 (0%)	6 (14%)
3	1 (1.7%)	0 (0%)	1 (2.3%)
not available	28	4	24
Survival status at data-cutoff			
alive	31 (36%)	7 (37%)	24 (36%)
dead	55 (64%)	12 (63%)	43 (64%)
Cause of death^1^			
disease related	32 (67%)	6 (55%)	26 (70%)
therapy related	6 (13%)	2 (18%)	4 (11%)
disease- and therapy related	9 (19%)	3 (27%)	6 (16%)
other cause	1 (2.1%)	0 (0%)	1 (2.7%)
not available	7	1	30
MM type			
intact Ig myeloma	65 (75.6%)	11 (57.9%)	54 (80.6%)
light-chain myeloma	18 (20.9%)	8 (42.1%)	10 (14.9%)
non-secretory myeloma	3 (3.5%)	0 (0%)	3 (4.5%)
Biopsy of EMD lesion performed	53 (62%)	15 (79%)	38 (57%)
Median time to EMD occurrence from MM diagnosis (months)	25.5 (7.0–52.0) [0.0–287.0]	N/A^9^	35.0 (20.0–72.0) [3.0–287.0]
Number of EMD lesions			
1	8 (9.3%)	3 (16%)	5 (7.5%)
2–5	23 (27%)	3 (16%)	20 (30%)
> 5	55 (64%)	13 (68%)	42 (63%)
Sites of organ involvement^2^			
lymph node	36 (42%)	9 (47%)	27 (40%)
cutaneous tissue	21 (24%)	3 (16%)	18 (27%)
retroperitoneal space	20 (23%)	4 (21%)	16 (24%)
muscle	18 (21%)	4 (21%)	14 (21%)
liver	18 (21%)	3 (16%)	15 (22%)
central nervous system	14 (16%)	1 (5.3%)	13 (19%)
pulmonary	9 (10%)	2 (11%)	7 (10%)
PCL			
EMD with concurrent PCL	12 (14%)	1 (5.3%)	11 (16%)
PCL at first diagnosis of MM	6 (7.0%)	1 (5.3%)	5 (7.5%)
ISS stage at initial diagnosis of MM^1^			
I	16 (24%)	4 (27%)	12 (23%)
II	25 (37%)	5 (33%)	20 (38%)
III	27 (40%)	6 (40%)	21 (40%)
not available	18	4	14
R-ISS stage at initial diagnosis of MM^1^			
I	7 (12%)	1 (7.1%)	6 (13%)
II	32 (54%)	8 (57%)	24 (53%)
III	20 (34%)	5 (36%)	15 (33%)
not available	27	5	22
Cytogenetic risk category			
standard risk	42 (55%)	9 (50%)	33 (57%)
high-risk^3^	34 (45%)	9 (50%)	25 (43%)
not available	10	1	9
del(17p)	26 (34%)	8 (44%)	18 (31%)
not available	9	1	8
t(4:14)	8 (11%)	2 (12%)	6 (10%)
not available	11	2	9
t(14;16)	3 (4.1%)	0 (0%)	3 (5.2%)
not available	12	3	9
t(14;20)	0 (0%)	0 (0%)	0 (0%)
not available	13	3	10
t(11;14)	15 (20%)	4 (25%)	11 (19%)
not available	12	3	9
gain/amp(1q)	24 (32%)	4 (25%)	20 (34%)
not available	11	3	8
del(1p)	7 (9.6%)	1 (6.3%)	6 (11%)
not available	13	3	10
bone marrow infiltration at first diagnosis of MM (%)^1^	56.0 (30.0–80.0) [0.0–100.0]	35.0 (17.0–80.0) [0.0–100.0]	63.0 (36.0–80.0) [8.0–100.0]
not available	15	3	12
bone marrow infiltration at occurrence of EMD (%)^1^	35.0 (6.5–80.0) [0.0–100.0]	35.0 (17.0–80.0) [0.0–100.0]	35.0 (3.0–80.0) [0.0–100.0]
not available	37	3	34
LOT prior to EMD^4^	2.0 (1.0–3.0) [0.0–11.0]	0	3.0 (1.0–4.0) [1.0–11.0]
triple-class exposed^5^	24 (28%)	0	24 (36%)
triple-class refractory^6^	16 (19%)	0	16 (24%)
penta-drug exposed^7^	11 (13%)	0	11 (16%)
penta-drug refractory^8^	9 (10%)	0	9 (13%)

Values are presented as median (interquartile range) [range] or as *n* (%), unless otherwise indicated.^1^ Percentages calculated among patients with available data.^2^ Percentages may exceed 100% as patients could have multiple sites of organ involvement.^3^ High-risk cytogenetics defined as del(17p), t(4;14), and t(14;16) according to R-ISS criteria.^4^ Lines of therapy administered prior to the occurrence of EMD.^5^ Triple-class exposed defined as prior exposure to a proteasome inhibitor (PI), an immunomodulatory drug (IMiD), and an anti-CD38 monoclonal antibody (mAb).⁶ Triple-class refractory defined as refractory to one PI, one IMiD, and one anti-CD38 monoclonal antibody.⁷ Penta-drug exposed defined as prior exposure to ≥ 2 PIs, ≥ 2 IMiDs, and one anti-CD38 monoclonal antibody.⁸ Penta-drug refractory defined as refractory to ≥ 2 PIs, ≥ 2 IMiDs, and one anti-CD38 monoclonal antibody.^9^ *N/A *not applicable

**Fig. 2:**
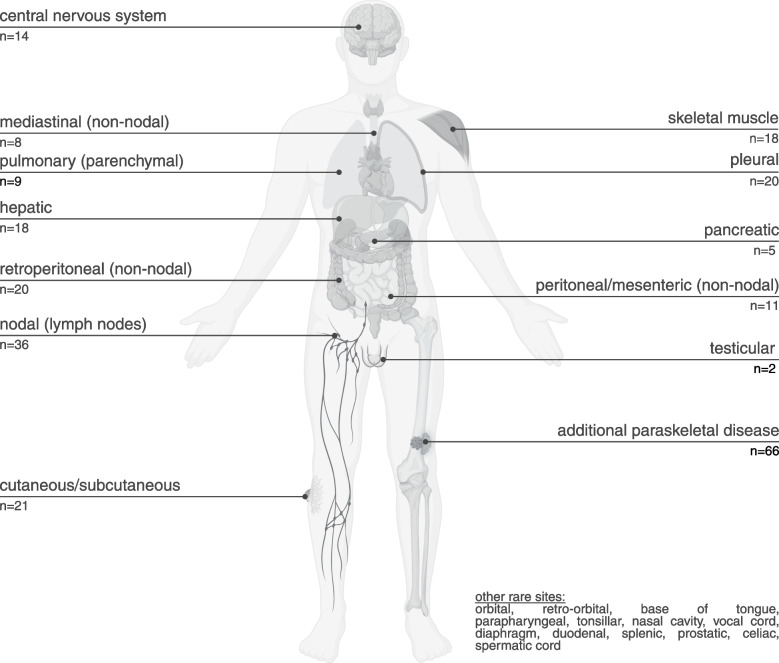
Anatomical distribution of EMD in the study cohort. For each localization, the total number of affected patients is shown. Additional paraskeletal lesions are displayed separately. Rare sites observed in single cases are listed below. Created with BioRender.com

Most patients (64%, *n* = 55) presented with multiple lesions (> 5), while 27% (*n* = 23) had 2–5 lesions, and 9% (*n* = 8) presented with a single lesion. The most frequent sites of organ involvement were lymph nodes (42%), cutaneous tissue (24%), retroperitoneal space (23%), muscle (21%), and liver (21%), followed by the CNS (16%). Organ involvement patterns were broadly similar between de novo and secondary EMD (data not shown). An overview of the anatomical distribution of EMD lesions is shown in Fig. 2.

Imaging modalities used for the detection of EMD reflected routine clinical practice. At initial diagnosis of EMD, CT was performed in 87% of patients, MRI in 73%, PET/CT in 44%, and bone scintigraphy in 2%; percentages exceed 100% as patients may have received more than one imaging modality. During follow-up and response assessment, CT was available in 84% of patients, MRI in 63%, and PET/CT in 39%. Regarding lymph node involvement (*n* = 36) as the most frequent site of organ involvement, 10 patients (28%) had histopathological confirmation (8 biopsy only, 2 biopsy plus PET/CT). PET/CT without biopsy was available in 17 patients (47%). In 7 patients (19%), diagnosis was based on CT/MRI and supported by follow-up imaging. In 2 patients (6%), diagnosis was based on cross-sectional imaging only without additional histopathological, PET/CT, or follow-up imaging confirmation.

**Fig. 3:**
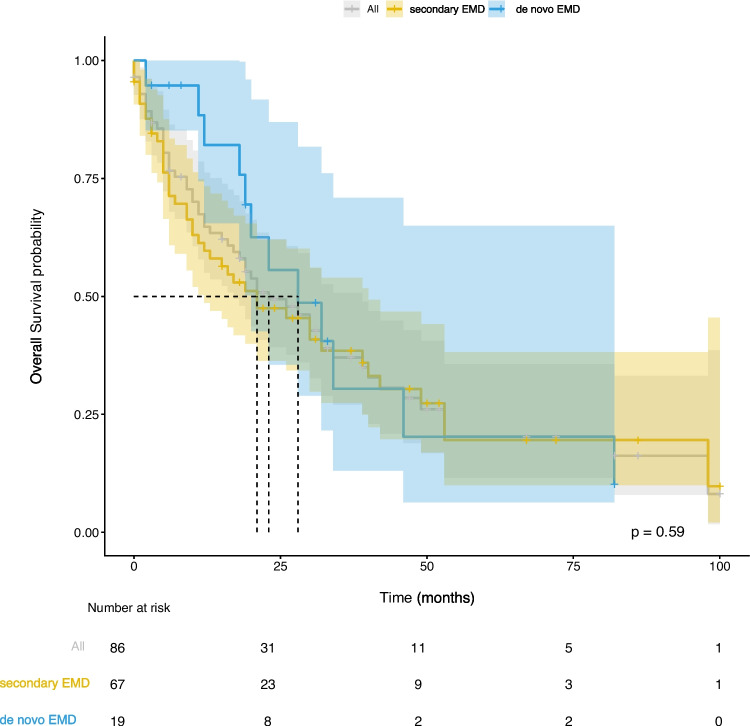
Overall survival from EMD diagnosis. Kaplan–Meier curves illustrating overall survival from the time of EMD diagnosis (OS-EMD) for the entire cohort (*n* = 86), patients with secondary EMD (*n* = 67), and de novo EMD (*n* = 19). Numbers at risk are shown below the plot. No statistically significant difference in OS-EMD was observed between the groups (*p* = 0.59)

The median follow-up for this cohort was 96 months (95% CI, 81–159). At the data cut-off, 55 patients (64%) had died, primarily due to disease progression (67% of deaths). The mOS for the entire cohort (*n* = 86) from initial MM diagnosis was 55 months (95% CI, 46–106), and the mOS-EMD was 23 months (95% CI, 17–39). Patients presenting with de novo EMD (*n* = 19) had a mOS-EMD of 28 months (95% CI, 20–NR) versus 21 months (95% CI, 12–40) for those with secondary EMD (*n* = 67); this difference was not statistically significant (*p* = 0.59) (Fig. 3). Patients with EMD and concurrent PCL (*n* = 12) showed a significantly inferior mOS of 17 months (95% CI, 16–NR; *p* = 0.0079) compared with 65 months (95% CI, 47–112) in those with EMD without PCL (*n* = 74). When survival was assessed from the time of EMD occurrence, outcomes were even shorter in patients with concurrent PCL (*n* = 12), with a mOS-EMD of only 9 months (95% CI, 3–NR; *p* = 0.002), whereas patients without concurrent PCL (*n* = 74) had a mOS-EMD of 30 months (95% CI, 19–42) (Supplementary Figure S1). Stratification by the number of prior lines of therapy (pLOT) in patients with EMD without PCL showed variation in mOS-EMD without a clear trend: patients with one prior line (*n* = 15) had a mOS-EMD of 30 months (95% CI, 15–NR), those with two to three prior lines (*n* = 23) had 40 months (95% CI, 13–NR), and those with ≥ 4 prior lines (*n* = 18) had 15 months (95% CI, 6–53) (*p* = 0.56) (data not shown). No significant differences in baseline characteristics or overall survival were observed between patients with biopsy-confirmed EMD and those diagnosed based on imaging alone (*p* = 0.9). However, patients without biopsy confirmation had received more prior lines of therapy at the time of EMD occurrence (median: 3 vs. 1; *p* = 0.005) and were more frequently penta-refractory (21% vs. 4%; *p* = 0.011). A comprehensive comparison is provided in Supplementary Table 1.

**Fig. 4:**
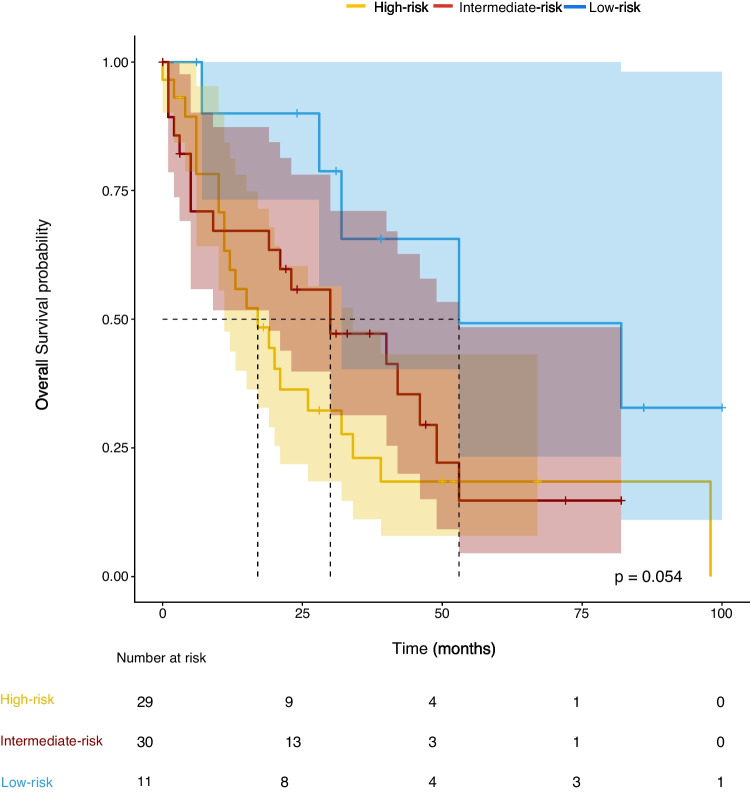
Overall survival from EMD diagnosis according to an exploratory anatomical site-based grouping. Kaplan–Meier curves illustrating overall survival from the time of EMD diagnosis according to anatomical risk groups: high-risk (HR; CNS, pulmonary, or retroperitoneal involvement; *n* = 29), intermediate-risk (IR; hepatic, muscular, or cutaneous involvement; *n* = 30), and low-risk (LR; lymph node involvement only; *n* = 11). A continuous gradient in survival was observed across the three groups, with patients in the HR category demonstrating a trend toward inferior median OS (mOS) compared with the IR and LR groups (17 vs. 30 vs. 53 months; *p* = 0.054)

Because concurrent PCL showed a significant adverse impact on OS, all site-specific survival analyses were restricted to patients without concurrent PCL to avoid confounding. To evaluate whether involvement of specific anatomical sites of EMD affected prognosis, we compared survival outcomes for the most frequently affected sites with all other EMD locations. Retroperitoneal involvement showed the largest absolute difference in mOS-EMD (17 months, 95% CI 11–NR vs. 30 months, 95% CI 21–98; *n* = 17), but this comparison was not statistically significant (p = 0.25). CNS involvement and pulmonary EMD had numerically shorter mOS-EMD with non-significant comparisons (19 months, 95% CI 13–NR vs. 30 months, 95% CI 20–46; *n* = 9; *p* = 0.69; 20 months, 95% CI 10–NR vs. 30 months, 95% CI 21–46; *n* = 8; *p* = 0.70, respectively). Cutaneous (mOS-EMD, 26 months, 95% CI 17–NR vs. 30 months, 95% CI 19–98; *n* = 16; *p* = 0.46), hepatic (30 months, 95% CI 17–NR vs. 28 months, 95% CI 19–49; *n* = 16; *p* = 0.91), and muscular involvement (mOS-EMD, 29 months, 95% CI 19–NR vs. 30 months, 95% CI 17–46; *n* = 15; *p* = 0.55) showed no trend towards survival differences. Lymph node involvement was associated with significantly better outcomes, with patients exhibiting an mOS-EMD of 42 months (95% CI 30–NR) compared with 19 months (95% CI 11–32) for those with EMD at other sites (*n* = 33; p = 0.011) (Supplementary Figures S2A–G). Based on these findings, we exploratory stratified patients into three prognostic subgroups. First, CNS, pulmonary, and retroperitoneal involvement were classified as high-risk EMD (HR; *n* = 29). Patients with HR lesions exhibited a near-significant trend toward shorter survival from EMD occurrence compared with those without HR involvement (17 months, 95% CI 11–34 vs. 40 months, 95% CI 28–53; *p* = 0.057). Next, we defined a low-risk EMD group characterized by lymph node involvement only (LR; *n* = 11). Comparing HR vs. LR EMD revealed a significant difference in mOS from EMD occurrence (17 months, 95% CI 11–34 vs. 53 months, 95% CI 32–NR; *p* = 0.015) (Supplementary Figure S3). To further explore anatomical differences in outcome, we then evaluated an exploratory three-tiered risk grouping within our cohort. Based on our findings, patients with hepatic, muscular, or cutaneous involvement were categorized as intermediate-risk (IR, *n* = 30), whereas lymph node involvement defined the LR group (*n* = 11); patients without involvement of any of these anatomical sites were excluded from this exploratory analysis. Comparison across these groups demonstrated a stepwise decline in survival outcomes, with a numerically shorter, but non-significant mOS from EMD in the HR group (17 months, 95% CI 11–34 vs. 30 months, 95% CI 19–53 vs. 53 months, 95% CI 32–NR; *p* = 0.054; Fig. 4). The total number of EMD lesions did not significantly influence OS (*p* = 0.67).

Furthermore, we assessed whether survival outcomes improved over time. Among patients with EMD without concurrent PCL (*n* = 74), OS did not improve across diagnostic year categories (before 2010, 2010–2014, 2015–2019, 2020–2024) and no survival advantage was observed for patients diagnosed in more recent years (data not shown).

Patients with EMD and concurrent PCL (*n* = 12) exhibited several distinct high-risk features compared with those without PCL (*n* = 74). They were significantly younger at the first occurrence of EMD (median age, 53.5 vs. 62.0 years; *p* = 0.038) and showed a markedly higher frequency of CNS involvement (42% vs. 12%; *p* = 0.023). 4 of 5 patients developed CNS involvement after the onset of PCL. A comprehensive comparison of patient characteristics is shown in Supplementary Table 2. Due to the small sample size, no further survival analyses were performed in this subgroup.

Overall, treatment approaches were highly heterogeneous, encompassing more than 50 distinct therapeutic regimens across 430 evaluated treatment lines, including all lines of therapy administered before and after EMD occurrence, of the 86 included patients (median 4.5 LOTs per patient, range 1–13). Among patients with *secondary* EMD, 4 did not receive active treatment due to poor performance status and were managed with best supportive care (BSC) only. Radiologic response assessments were available for 46% of LOTs in patients with EMD. Intrathecal therapy was administered in 6 cases.

Radiotherapy was performed in 79 of 430 LOTs (18%). In 38 of these 79 LOTs (48%), radiotherapy was applied to sites consistent with PSD – predominantly intraspinal/epidural extensions. Osteolytic bone lesions were irradiated in 36 of 79 LOTs (45%), mainly for pain control or skeletal stabilization. True EMD manifestations were irradiated in 18 of 79 LOTs (23%). The CNS (brain or spinal axis) represented the most frequent site of EMD irradiation (*n* = 8, 10%), followed by cutaneous/subcutaneous/muscular tissue involvement (*n* = 5, 6%) and lymph nodes or mediastinal involvement (*n* = 4, 5%). Single cases of abdominal (*n* = 1) and hepatic (*n* = 1) involvement were also irradiated. Notably, individual LOTs could include irradiation of multiple anatomical sites simultaneously.

In patients presenting with EMD at initial diagnosis (*n* = 19), the majority (n = 10, 53%) underwent induction followed by HDT/ASCT (*n* = 8 without and *n* = 2 with CD38 antibody-based induction), achieving an ORR of 100% post HDT/ASCT, including 38% of cases assessed by serology-only response. Non-transplant regimens included CD38 antibody-based regimens (*n* = 4) or cyclophosphamide-based regimens (*n* = 2). Across subsequent treatment lines, regimens were highly heterogeneous. Selected patients achieved responses with repeat HDT/ASCT, alloSCT, or PACE (cisplatin, doxorubicin, cyclophosphamide, etoposide). Novel therapies including CAR T-cell therapy and bsAb were administered only in later lines and demonstrated variable activity (data not shown). ORR declined with increasing numbers of treatment lines (≥ 4 lines; data not shown). First-line treatment regimens in patients with de novo EMD are summarized in Table 2.

**Table 2:** First treatment line and outcomes in patients with de novo EMD

First treatment line initiated in de novo EMD	*n*	ORR^1^ (%)	CR (seCR^2^)	VGPR (seVGPR^2^)	PR (sePR^2^)	N/A^3^ (≥ PR)	No response^4^
all	**19**	**15 (79)**	**2 (1)**	**2 (3)**	**3 (2)**	**2**	**4**
HDT/ASCT w CD38	8	8 (100)	1(1)	2(2)	2 (0)	0	0
CD38-based	4	3 (75)	0 (0)	0 (0)	1 (1)	1	1
HDT/ASCT w/o CD38	2	2 (100)	1(0)	0 (1)	0 (0)	0	0
Cyclophosphamide-based	2	0 (0)	0 (0)	0 (0)	0 (0)	0	2
PI-based doublet	1	1 (100)	0 (0)	0 (0)	0 (1)	0	0
Other	2	1 (50)	0 (0)	0 (0)	0 (0)	1	1

^1^ Defined as PR or better; ^2^ response assessment based solely on serological parameters (imaging not performed); ^3^ N/A = response documented but not classifiable; ^4^ includes MR, SD, PD

Abbreviations: *CD38-based* anti-CD38 antibody–based therapy (daratumumab, isatuximab), *HDT/ASCT* high-dose melphalan with autologous stem-cell transplantation, *PI-based* proteasome inhibitor–based therapy, *w CD38* with CD38 antibody–based induction, *w/o CD38* without CD38 antibody–based induction

In the first treatment line initiated after EMD occurrence (*n* = 63; 4 received BSC), treatment approaches and outcomes were highly heterogeneous, with an ORR of 52% (including 39% of cases assessed by serology-only response) and nearly half of patients being primarily refractory. The most frequently used regimens included carfilzomib-based therapy (*n* = 12), CD38 antibody-based regimens (*n* = 7) and PACE-based chemotherapy (*n* = 7). HDT/ASCT (*n* = 5) and alloSCT (*n* = 5) achieved the highest response rates. CAR T-cell therapy demonstrated promising activity (*n* = 4), while monotherapy with bsAb showed no activity in this setting (*n* = 3). In subsequent treatment lines for relapsed or recurrent EMD (i.e., all lines beyond the first post-EMD line), outcomes remained poor across most conventional regimens, whereas novel therapies continued to show activity (CAR T: *n* = 6; ORR 100%; bsAb: *n* = 11; ORR 45%, data not shown). Similar to de novo EMD, ORR declined markedly in later lines of therapy (≥ 4 lines; data not shown). First-line treatment regimens and response outcomes following the occurrence of secondary EMD are summarized in Table 3.

**Table 3:** First treatment line and outcomes in patients after the occurrence of secondary EMD

First treatment line initiated after occurrence of secondary EMD	*n*	ORR^1^ (%)	CR (seCR^2^)	VGPR (seVGPR^2^)	PR (sePR^2^)	N/A^3^ (≥ PR)	No response^4^
all	**63**	**33 (52)**	**4 (3)**	**5 (3)**	**7 (7)**	**4**	**30**
Carfilzomib-based	12	4 (33)	0(0)	1 (0)	2 (0)	1	8
CD38-based	7	4 (57)	1(0)	0 (0)	1 (2)	0	3
PACE	7	4 (57)	0(0)	1 (0)	1 (2)	0	3
AlloSCT	5	3 (60)	0 (0)	1 (1)	1 (0)	0	2
HDT/ASCT	5	5 (100)	0 (1)	2 (0)	1 (1)	0	0
other	7	3 (43)	1 (0)	0 (0)	0 (1)	1	4
CAR T	4	4 (100)	2 (1)	0 (1)	0 (0)	0	0
Elotuzumab-based (EloRd/EloPd)	4	1 (25)	0 (0)	0 (1)	0 (0)	0	3
BsAb	3	0 (0)	0 (0)	0 (0)	0 (0)	0	3
IMiD-based doublet (Rd)	3	2 (67)	0 (1)	0 (0)	1 (0)	0	1
Poma-based^5^	2	2 (100)	0 (0)	0 (0)	0 (0)	2	0
PI-based doublet	2	1 (50)	0 (0)	0 (0)	0 (1)	0	1
Cyclophosphamide-based	2	0 (0)	0 (0)	0 (0)	0 (0)	0	2

^1^ Defined as PR or better; ^2^ response assessment based solely on serological parameters (imaging not performed); ^3^ N/A = response documented but not classifiable; ^4^ includes MR, SD, PD; ^5^ except EPd and PCd

Abbreviations: *AlloSCT* allogeneic stem cell transplantation (conditioning regimens varied by center), *BsAb* bispecific antibody (ABBV-383, talquetamab, teclistamab, TNB-383B, elranatamab), *CAR T* chimeric antigen receptor T-cell therapy (idecabtagene vicleucel, ciltacabtagene autoleucel), *CD38-based* anti-CD38 antibody–based therapy (daratumumab, isatuximab), *EloPd* elotuzumab–pomalidomide–dexamethasone, *EloRd* elotuzumab–lenalidomide–dexamethasone, *HDT/ASCT* high-dose melphalan with autologous stem-cell transplantation, *IMiD-based* immunomodulatory drug–based therapy, *PACE* cisplatin–doxorubicin–cyclophosphamide–etoposide, *PI-based* proteasome inhibitor–based therapy, *Poma-based* pomalidomide-based therapy, *Rd* lenalidomide–dexamethasone

As radiologic response assessment was not available in all cases, serology-only responses were documented separately in both tables indicated by the prefix 'se' preceding the response category.

To further characterize treatment outcomes, PFS was analyzed in an exploratory line-level analysis across 216 treatment lines initiated from the time of EMD occurrence onwards in 82 patients. Median PFS declined progressively with increasing lines of therapy: in the first-line setting, median PFS was 5 months (95% CI: 3–10 months; *n* = 82 treatment lines; 69 events); in the 1st–3rd relapse setting (lines 2–4), median PFS was 3 months (95% CI: 2–5 months; *n* = 113 treatment lines; 95 events); and beyond the 3rd relapse (≥ 4th relapse), median PFS was 1 month (95% CI: 1–4 months; *n* = 23 treatment lines; 22 events; *p* = 0.0028) (Supplementary Figure S4).

Regarding OS from initial MM diagnosis, univariate analysis showed that age at initial diagnosis (hazard ratio (HR) 1.03, 95% CI 1.00–1.06, *p* = 0.034), ECOG 2 (HR 4.87, 95% CI 1.43–16.58, *p* = 0.011), ISS III (HR 2.73, 95% CI 1.19–6.29, *p* = 0.018), R-ISS III (HR 4.04, 95% CI 1.25–13.03, *p* = 0.019), high-risk cytogenetics (HR 1.78, 95% CI 1.00–3.16, *p* = 0.050), and primary refractoriness (HR 3.28, 95% CI 1.50–7.14, *p* = 0.003) were significantly associated with poorer survival. However, in the full multivariable Cox model including all candidate covariates (*n* = 38, 26 events; 48 patients excluded due to missing covariate data), as well as in the reduced multivariable analysis (*n* = 39, 27 events; 47 patients excluded due to missing covariate data), none of these parameters independently predicted shorter OS. Regarding OS from first occurrence of EMD, univariable Cox analysis revealed lymph node involvement to be associated with superior outcomes (HR 0.46, 95% CI 0.26–0.82, *p* = 0.008). This association remained significant in the full multivariable Cox model including all candidate covariates (*n* = 86, 55 events; HR 0.45, 95% CI 0.24–0.85, *p* = 0.014) as well as in the reduced multivariable model including lymph node involvement (*n* = 86, 55 events; HR 0.46, 95% CI 0.26–0.82, *p* = 0.008).

## Discussion

In this retrospective, single-center real-world analysis, we present a well-characterized cohort of patients with radiologically confirmed true EMD in MM, which represents an independent high-risk feature associated with inferior OS [3, 18, 19, 22, 34]. Given the highly heterogeneous treatment approaches observed in our cohort and the lack of evidence-based treatment recommendations, our goal was to determine potential risk factors and to optimize treatment decisions for this high-risk population.

First, the analyzed cohort exhibited an enrichment of high-risk cytogenetic features, known to be associated with worse outcomes, regardless of EMD. In our cohort, high-risk cytogenetic features were associated with inferior OS compared with standard-risk cytogenetics in univariate analysis, but did not retain independent prognostic significance in multivariate analysis, which may reflect limited statistical power due to the relatively small sample size of the cohort. Interestingly, del(17p) was the most frequent alteration (de novo EMD: 44%, secondary EMD: 31%), and substantially higher than the 5–10% reported in patients with newly diagnosed MM without extramedullary spread [35, 36]. This aligns with a recent retrospective analysis demonstrating both an enrichment of del(17p) among EMD patients (31.3% vs. 16.4%) and its association with inferior survival [20].

Second, we demonstrate that in our cohort involvement of specific anatomical sites influences clinical outcomes. Notably, lymph node involvement was associated with significantly longer OS compared with all other sites in our cohort (*p* = 0.011), though this finding should be interpreted with caution given the exploratory nature of the analysis and the limited sample size. Moreover, in the hypothesis-generating anatomical risk grouping, patients classified within the high-risk EMD group, characterized by CNS, retroperitoneal, and/or pulmonary involvement, exhibited significantly shorter OS than the low-risk EMD group, which was defined by lymph node involvement only.

Our clinical findings align with recent spatial multi-omics studies revealing pronounced inter- and intralesional heterogeneity of EMD lesions. This heterogeneity is observed in the genetic composition of malignant PC, including copy-number variation and biallelic inactivation of tumor-suppressor genes, and in the tumor microenvironment, which, among other features, shows enrichment of exhausted T cells [37–39]. A recent retrospective analysis reported distinct TP53 mutation sites between EMD and non-EMD patients, consistent with the heterogeneity of PC subclones [20]. Additionally, some organs may provide immune-privileged environments, as well described for the CNS, which could facilitate immune escape and treatment resistance [3, 40].

Together, these findings suggest that EMD subclones growing within specific anatomical niches, interacting with the local microenvironment, may possess more aggressive biological features, potentially contributing to poorer therapeutic responses and adverse clinical outcomes. Accordingly, the anatomical site of EMD could be considered in treatment selection. The total number of lesions did not influence survival, further supporting the notion that tumor biology and site-specific microenvironmental interactions, rather than overall disease burden, may be more relevant determinants of prognosis in EMD.

Besides this, we identified 14% of patients presenting with concurrent PCL, a frequency substantially higher than the approximately 6% typically reported for primary and 1% for secondary PCL in unselected MM cohorts [41]. This may indicate shared pathophysiological mechanisms driving BM escape. In their BM–resident state, CXCR4–CXCL12 interactions mediate homing and retention of MM cells within specialized BM niches, whereas loss of CXCR4 and other chemokine receptors (e.g., CCR2), along with decreased expression of adhesion molecules such as CD56, facilitates egression, with loss of CD56 in particular representing a common biological pathway linking EMD and PCL and supporting the concept of a biological continuum between these entities [8, 15, 42]. Considering that our cohort was derived from a tertiary referral center, a potential selection bias toward biologically aggressive cases cannot be fully excluded. Among patients with concurrent PCL (*n* = 12), CNS involvement was more frequent than in those without (42% vs. 12%; *p* = 0.023). This subgroup is small, and the comparison should be interpreted as exploratory. However, this finding is consistent with previous reports describing CNS relapse in the context of PCL, as well as an increased prevalence of PCL among patients with CNS myeloma, with about 10–20% presenting with concurrent PCL [43–47]. The high rate of coexistence of CNS involvement and PCL likely reflects a markedly aggressive tumor phenotype enabling efficient dissemination of malignant plasma cells.

Evaluating treatment regimen efficacy, our data demonstrate no significant improvement in OS across diagnostic periods, suggesting that outcomes in patients with EMD remain unsatisfactory despite the introduction of modern therapeutic agents. At first glance this may seem surprising, but is in line with previous findings reported by Zanwar et al. [14].

When comparing T-cell–redirecting therapies, we observed the highest ORRs with CAR T-cell therapy, even in heavily pretreated patients. BsAb showed activity, but ORR was markedly lower than with CAR T-cell therapy. Findings from a multicenter, retrospective analysis of patients treated with idecabtagene vicleucel (ide-cel) indicate lower ORR of CAR T-cell therapy in patients with EMD compared to patients without EMD (ORR 52% vs. 82%). Similarly, in pivotal trials of bsAbs, the presence of EMD was associated with substantially reduced efficacy: for the GPRC5D-targeted bsAb talquetamab in MonumenTAL-1, ORRs of 41–48% were observed in patients with EMD compared with 69–74% overall, and for the BCMA-targeted bsAb teclistamab in MajesTEC-1, an ORR of 35.7% was reported in patients with EMD compared with 63% overall [48, 49].

Nevertheless, CAR T-cell therapy should be considered as CARTITUDE-4 showed superior PFS and OS for patients with EMD treated with ciltacabtagene autoleucel (cilta-cel) compared with the SOC control arm [50–52].

Recent prospective studies have specifically addressed treatment in patients with MM and EMD. Dual-antigen targeting with teclistamab plus talquetamab, evaluated in the phase 2 RedirecTT-1 trial, demonstrated encouraging activity in patients with RRMM and EMD, with synchronous targeting of two major therapeutic myeloma antigens resulting in a high ORR of 79%, and therefore constitutes another treatment option [30]. The prospective phase II EMN19 study evaluated DaraVCD in 40 patients with newly diagnosed or first-relapse MM and extramedullary plasmacytomas, reporting an ORR of 80% and a median PFS of 25.8 months [53]; EMN19 included both true EMD and paraskeletal disease without separate subgroup reporting, which limits direct comparability with cohorts restricted to true EMD. Regarding EMD-directed approaches in NDMM, a recent prospective phase 2 trial evaluated selinexor combined with bortezomib, lenalidomide, and dexamethasone (SVRD) in 29 patients with NDMM and EMD, reporting an ORR of 89.7% and complete EMD resolution in 79.3% of patients, with a 12-month PFS of 87.9% [54]. These results suggest that the addition of selinexor to induction therapy may represent a rational frontline approach for EMD-positive NDMM, though randomized confirmation is warranted.

In summary, the available data indicate that although novel T-cell–redirecting therapies, particularly dual-antigen targeting strategies, show activity in EMD, they remain limited in their ability to fully overcome the aggressive biology of the disease, highlighting EMD as a persistent area of major unmet clinical need.

A key limitation of this study is its single-center design with a relatively small and heterogeneous cohort, which limits the generalizability of the conclusions but provides insights into the current treatment landscape of a real-world cohort in Germany. Furthermore, as this study focused exclusively on patients with radiologically confirmed EMD, no internal control cohort of patients without EMD was available for comparison, which limits the ability to directly quantify the prognostic impact of extramedullary involvement relative to bone marrow-only disease. In addition, data were collected from institutional radiology databases by looking for keywords. As radiologic reports were not uniformly standardized and advanced imaging modalities such as PET/CT or whole-body MRI were not consistently available, misclassification of affected patients as non-EMD and selection bias cannot be fully excluded. Staging examinations that were conducted in other radiology practices or hospitals were not available for our analyses. Furthermore, the cohort represents a population treated at a tertiary referral care center and is therefore likely enriched for patients with aggressive or refractory disease. As imaging-based response assessment was available for only 46% of treatment lines, response assessment was based solely on serologic parameters for a subset of patients. This represents a key limitation of the present study, and comparisons of treatment responses across regimens must therefore be interpreted with caution. Furthermore, comparative interpretation of treatment efficacy across regimens is limited by substantial selection bias, as treatment selection was heavily influenced by patient fitness, prior therapy exposure, and disease stage. In the exploratory line-level PFS analysis, individual patients contributed multiple treatment lines, resulting in non-independent observations; therefore, results should be interpreted descriptively. Owing to the small number of cases per subgroup, detailed pairwise comparisons of OS by anatomical site were precluded; therefore, only comparisons between single sites and all other sites were performed. Given the limited sample size and potential confounding by treatment history, these analyses are descriptive in nature. Interpretation of the favorable outcomes associated with lymph node involvement is limited by heterogeneous diagnostic confirmation, with incomplete histopathological or PET/CT verification. However, sensitivity analyses showed no survival difference between biopsy-confirmed and imaging-defined cases. In addition, the three-tiered anatomical risk grouping was developed and evaluated within the same dataset and therefore represents a cohort-specific, hypothesis-generating approach rather than a validated clinical stratification tool and requires external validation. Nevertheless, the observed associations appear biologically plausible. Finally, multivariable analyses were limited by missing covariate data and the relatively small sample size and should therefore be interpreted cautiously.

To enhance future patient care, standardization of diagnostic approaches is essential to ensure consistent disease assessment and data reproducibility across studies and in routine clinical care. Implementation of functional imaging with ^1^⁸F-FDG PET/CT represents a critical step toward uniform response evaluation [55]. Imaging-based minimal residual disease (MRD) assessment should complement BM-based MRD assessment, as residual EMD lesions may persist despite BM MRD negativity and are associated with inferior outcomes [55, 56]. To better characterize the distinct tumor biology of extramedullary lesions and their subclonal heterogeneity, longitudinal translational research efforts with sequential tissue sampling throughout the disease course are required to capture tumor evolution. This may help to decipher the biological drivers of extramedullary dissemination and resistance mechanisms, and to identify potential molecular targets for future therapies [37–39].

In conclusion, our data provide insights into a high-risk population and reflect the real-world management of this distinct subentity of MM. The scarcity of large prospective clinical studies and the absence of clear consensus recommendations remain major limitations in the management of patients with EMD [15]. To date most available evidence derives from retrospective analyses and the definition of true EMD remains heterogeneous across studies, underscoring the need for universal disease and endpoint definitions provided by future guidelines.

Finally, in the absence of clinical trial data to guide evidence-based treatment approaches, it is crucial to establish national or international registries dedicated to EMD. This would represent a first step toward systematically collecting and disseminating data to better characterize disease presentation, diagnostic and treatment strategies, disease trajectories, and outcomes in this distinct MM subgroup.

## Supplementary Information

Below is the link to the electronic supplementary material.

Supplementary file1 (DOCX 24 KB)

Supplementary file2 (DOCX 31 KB)

Supplementary file3 Overall survival from EMD diagnosis by concurrent plasma cell leukemia.Kaplan–Meier curves illustrating overall survival from EMD (OS-EMD) in patients with EMD without concurrent PCL (*n* = 74) and with concurrent PCL (*n* = 12). OS-EMD differed significantly between the groups (*p* = 0.002). (PDF 28 KB)

Supplementary file4 Survival outcomes according to anatomical sites of EMD.Kaplan–Meier curves illustrating overall survival from the time of EMD diagnosis stratified by anatomical site (A–G): A, retroperitoneal; B, CNS; C, pulmonary; D, cutaneous; E, hepatic; F, muscular; G, lymph node involvement. (PDF 39 KB)

Supplementary file5 (PDF 78 KB)

Supplementary file6 (PDF 96 KB)

Supplementary file7 (PDF 78 KB)

Supplementary file8 (PDF 78 KB)

Supplementary file9 (PDF 26 KB)

Supplementary file10 (PDF 60 KB)

Supplementary file11 Overall survival from the time of EMD diagnosis according to anatomical high-risk and low-risk organ involvement. Kaplan–Meier curves comparing overall survival from EMD diagnosis (OS-EMD) between patients with high-risk EMD (HR; CNS, pulmonary, or retroperitoneal involvement; *n* = 29) and those with low-risk EMD (LR; lymph node involvement only; *n* = 11). Median OS-EMD was significantly shorter in the HR group compared with the LR group (17 vs. 53 months; *p* = 0.015). (PDF 32 KB)

Supplementary file12 Progression-free survival by line of therapy from EMD diagnosis.Kaplan–Meier curves illustrating progression-free survival (PFS) across treatment lines initiated from the time of EMD occurrence onwards (*n*=216 treatment lines in 82 patients). Groups are shown for newly diagnosed MM (NDMM), 1st-3rd relapse, and ≥4th relapse settings. Median PFS declined progressively with increasing lines of therapy (*p*=0.0028) (PDF 39 KB)

## Data Availability

The datasets generated and/or analyzed during the current study are available from the corresponding author on reasonable request.
