# Supplementary material for: Characteristics, treatment regimens, and outcomes of patients with true extramedullary multiple myeloma: a real-world monocentric analysis
Source: Ann Hematol. 2026 Jun 16;105(7):300. doi: 10.1007/s00277-026-07118-6 (PMC13272244; doi:10.1007/s00277-026-07118-6)
Supplement: Supplementary file 1 — Supplementary file1 (DOCX 24 KB) [file 277_2026_7118_MOESM1_ESM.docx]

**Supplementary Table 1. Baseline and disease characteristics stratified by biopsy confirmation status.**

| **Characteristic** | **Imaging-only EMD (N = 33)** | **Biopsy-confirmed EMD (N = 53)** | **p-value^4^** |
| --- | --- | --- | --- |
| **Sex** |  |  | 0.2 |
| male | 27 (82%) | 36 (68%) |  |
| female | 6 (18%) | 17 (32%) |  |
| **Age at initial diagnosis of MM (years)** | 55.0 (49.0–66.0) [39.0–80.0] | 57.0 (50.0–64.0) [37.0–75.0] | 0.8 |
| **ECOG at initial diagnosis of MM¹** |  |  | 0.8 |
| 0 | 7 (32%) | 13 (36%) |  |
| 1 | 12 (55%) | 19 (53%) |  |
| 2 | 2 (9.1%) | 4 (11%) |  |
| 3 | 1 (4.5%) | 0 (0%) |  |
| not available | 11 | 17 |  |
| **Survival status at data-cutoff** |  |  | >0.9 |
| alive | 12 (36%) | 19 (36%) |  |
| dead | 21 (64%) | 34 (64%) |  |
| **MM type** |  |  | 0.12 |
| intact Ig myeloma | 22 (67%) | 43 (81%) |  |
| light-chain myeloma | 9 (27%) | 9 (17%) |  |
| non-secretory myeloma | 1 (3.0%) | 2 (3.8%) |  |
| **EMD at initial diagnosis of MM** |  |  | 0.079 |
| yes | 4 (12%) | 15 (28%) |  |
| no | 29 (88%) | 38 (72%) |  |
| **Median time to EMD occurrence from MM diagnosis (months)** | 37.0 (22.0–98.0) [0.0–187.0] | 18.0 (1.0–45.0) [0.0–287.0] | 0.007 |
| **Number of EMD lesions** |  |  | 0.4 |
| 1 | 4 (12%) | 4 (7.5%) |  |
| 2–5 | 11 (33%) | 12 (23%) |  |
| >5 | 18 (55%) | 37 (70%) |  |
| **Sites of organ involvement²** |  |  |  |
| lymph node | 11 (33%) | 25 (47%) | 0.2 |
| cutaneous tissue | 8 (24%) | 13 (25%) | >0.9 |
| retroperitoneal space | 8 (24%) | 12 (23%) | 0.9 |
| muscle | 9 (27%) | 9 (17%) | 0.3 |
| liver | 6 (18%) | 12 (23%) | 0.6 |
| central nervous system | 2 (6.1%) | 12 (23%) | 0.043 |
| pulmonary | 3 (9.1%) | 6 (11%) | >0.9 |
| **Concurrent PCL** |  |  | 0.12 |
| yes | 2 (6.1%) | 10 (19%) |  |
| no | 31 (94%) | 43 (81%) |  |
| **ISS stage at initial diagnosis of MM¹** |  |  | 0.7 |
| I | 6 (21%) | 10 (25%) |  |
| II | 12 (43%) | 13 (33%) |  |
| III | 10 (36%) | 17 (43%) |  |
| not available | 5 | 13 |  |
| **R-ISS stage at initial diagnosis of MM¹** |  |  | >0.9 |
| I | 2 (9.5%) | 5 (13%) |  |
| II | 12 (57%) | 20 (53%) |  |
| III | 7 (33%) | 13 (34%) |  |
| not available | 12 | 15 |  |
| **Cytogenetic risk category** |  |  | 0.092 |
| standard risk | 19 (68%) | 23 (48%) |  |
| high-risk³ | 9 (32%) | 25 (52%) |  |
| not available | 5 | 5 |  |
| **del(17p)** |  |  | 0.2 |
| yes | 7 (24%) | 19 (40%) |  |
| absent | 22 (76%) | 29 (60%) |  |
| not available | 4 | 5 |  |
| **t(4;14)** |  |  | 0.7 |
| yes | 2 (7.1%) | 6 (13%) |  |
| absent | 26 (93%) | 41 (87%) |  |
| not available | 5 | 6 |  |
| **t(14;16)** |  |  | 0.3 |
| yes | 0 (0%) | 3 (6.5%) |  |
| absent | 28 (100%) | 43 (93%) |  |
| not available | 5 | 7 |  |
| **t(11;14)** |  |  | 0.14 |
| yes | 3 (11%) | 12 (26%) |  |
| absent | 24 (89%) | 35 (74%) |  |
| not available | 6 | 6 |  |
| **gain/amp(1q)** |  |  | 0.9 |
| yes | 9 (31%) | 15 (33%) |  |
| absent | 20 (69%) | 31 (67%) |  |
| not available | 4 | 7 |  |
| **del(1p)** |  |  | 0.2 |
| yes | 1 (3.7%) | 6 (13%) |  |
| absent | 26 (96%) | 40 (87%) |  |
| not available | 6 | 7 |  |
| **bone marrow infiltration at first diagnosis of MM (%)¹** | 50.0 (20.0–80.0) [9.0–100.0] | 70.0 (40.0–80.0) [0.0–100.0] | 0.2 |
| not available | 4 | 11 |  |
| **bone marrow infiltration at occurrence of EMD (%)¹** | 28.0 (5.5–59.5) [0.0–100.0] | 40.0 (6.5–80.0) [0.0–100.0] | 0.6 |
| not available | 17 | 20 |  |
| **Prior lines of therapy at EMD occurrence** | 3.0 (1.0–4.0) [0.0–11.0] | 1.0 (0.0–3.0) [0.0–9.0] | 0.005 |
| **Prior refractory status** |  |  | 0.011 |
| none | 18 (55%) | 43 (81%) |  |
| triple-class refractory | 8 (24%) | 8 (15%) |  |
| penta-drug refractory | 7 (21%) | 2 (3.8%) |  |
| **Treatment exposure at EMD occurrence** |  |  | 0.2 |
| none | 17 (52%) | 34 (64%) |  |
| triple-class exposed | 9 (27%) | 15 (28%) |  |
| penta-drug exposed | 7 (21%) | 4 (7.5%) |  |
| **Prior BCMA-targeted therapy** |  |  | 0.019 |
| yes | 4 (12%) | 0 (0%) |  |
| no | 29 (88%) | 53 (100%) |  |

Values are presented as median (interquartile range) [range] or as n (%), unless otherwise indicated.

¹ Percentages calculated among patients with available data.

² Percentages may exceed 100% as patients could have multiple sites of organ involvement.

³ High-risk cytogenetics defined as del(17p), t(4;14), and t(14;16) according to R-ISS criteria.

^4^ Wilcoxon rank sum test; Fisher's exact test; Pearson's Chi-squared test.
