# Supplementary material for: Characteristics, treatment regimens, and outcomes of patients with true extramedullary multiple myeloma: a real-world monocentric analysis
Source: Ann Hematol. 2026 Jun 16;105(7):300. doi: 10.1007/s00277-026-07118-6 (PMC13272244; doi:10.1007/s00277-026-07118-6)
Supplement: Supplementary file 2 — Supplementary file2 (DOCX 31 KB) [file 277_2026_7118_MOESM2_ESM.docx]

|  | **EMD without concurrent PCL**  (N = 74) | ***EMD with concurrent PCL*** (N = 12) | ***p-value^4^*** |  |
| --- | --- | --- | --- | --- |
| **Sex** |  |  | 0.013 |  |
| male | 58 (78%) | 5 (42%) |  |  |
| female | 16 (22%) | 7 (58%) |  |  |
| **Age at initial diagnosis of MM (years)** | 58.5 (50.0–66.0) [39.0–80.0] | 51.0 (46.0–61.5) [37.0–75.0] | 0.12 |  |
| **ECOG at initial diagnosis of MM^1^** |  |  | 0.091 |  |
| 0 | 20 (40%) | 0 (0%) |  |  |
| 1 | 24 (48%) | 7 (88%) |  |  |
| 2 | 5 (10%) | 1 (13%) |  |  |
| 3 | 1 (2.0%) | 0 (0%) |  |  |
| not available | 24 | 4 |  |  |
| **Survival status at data-cutoff** |  |  | >0.9 |  |
| alive | 27 (36%) | 4 (33%) |  |  |
| dead | 47 (64%) | 8 (67%) |  |  |
| **Cause of death^1^** |  |  | 0.4 |  |
| disease related | 25 (61%) | 7 (100%) |  |  |
| therapy related | 6 (15%) | 0 (0%) |  |  |
| disease- and therapy related | 9 (22%) | 0 (0%) |  |  |
| other cause | 1 (2.4%) | 0 (0%) |  |  |
| not available | 6 | 1 |  |  |
| **MM type** |  |  |  |  |
| intact Ig myeloma | 57 (77.0%) | 8 (66.7%) |  |  |
| light-chain myeloma | 18 (20.3%) | 3 (25.0%) |  |  |
| non-secretory myeloma | 2 (2.7%) | 1 (8.3%) |  |  |
| **Biopsy of EMD lesion performed** | 43 (58%) | 10 (83%) | 0.12 |  |
| **Median time to secondary EMD occurrence from MM diagnosis (months)** | 39.0 (25.0–88.5) [3.0–287.0] | 15.0 (9.0–28.0) [6.0–72.0] | <0.001 |  |
| **Number of EMD lesions** |  |  | 0.8 |  |
| 1 | 46 (62%) | 9 (75%) |  |  |
| 2-5 | 7 (9.5%) | 1 (8.3%) |  |  |
| >5 | 21 (28%) | 2 (17%) |  |  |
| **Sites of organ involvement^2^** |  |  |  |  |
| lymph node | | 33 (45%) | 3 (25%) | 0.2 |
| cutaneous tissue | 16 (22%) | 5 (42%) | 0.2 |  |
| retroperitoneal space | 17 (23%) | 3 (25%) | >0.9 |  |
| muscle | 15 (20%) | 3 (25%) | 0.7 |  |
| liver | 16 (22%) | 2 (17%) | >0.9 |  |
| central nervous system | 9 (12%) | 5 (42%) | 0.023 |  |
| pulmonary | 8 (11%) | 1 (8.3%) | >0.9 |  |
| **De novo EMD** | 18 (24%) | 1 (8.3%) | 0.3 |  |
| **PCL at first diagnosis of MM** | 0 (0%) | 6 (50%) |  |  |
| **ISS stage at initial diagnosis of MM^1^** |  |  | 0.14 |  |
| I | 15 (27%) | 1 (8.3%) |  |  |
| II | 22 (39%) | 3 (25%) |  |  |
| III | 19 (34%) | 8 (67%) |  |  |
| not available | 18 | 0 |  |  |
| **R-ISS stage at initial diagnosis of MM^1^** |  |  | 0.021 |  |
| I | 6 (13%) | 1 (8.3%) |  |  |
| II | 29 (62%) | 3 (25%) |  |  |
| III | 12 (26%) | 8 (67%) |  |  |
| not available | 27 | 0 |  |  |
| **Cytogenetic risk category** |  |  | 0.7 |  |
| standard risk | 36 (56%) | 6 (50%) |  |  |
| high-risk^3^ | 28 (44%) | 6 (50%) |  |  |
| not available | 10 | 0 |  |  |
| **del(17p)** | 21 (32%) | 5 (42%) | 0.5 |  |
| absent | 44 (68%) | 7 (58%) |  |  |
| not available | 9 | 0 |  |  |
| **t(4:14)** | 7 (11%) | 1 (8.3%) | >0.9 |  |
| absent | 56 (89%) | 11 (92%) |  |  |
| not available | 11 | 0 |  |  |
| **t(14;16)** | 2 (3.2%) | 1 (8.3%) | 0.4 |  |
| absent | 60 (97%) | 11 (92%) |  |  |
| not available | 12 | 0 |  |  |
| **t(14;20)** | 0 (0%) | 0 (0%) |  |  |
| absent | 61 (100%) | 12 (100%) |  |  |
| not available | 13 | 0 |  |  |
| **t(11;14)** | 12 (19%) | 3 (25%) | 0.7 |  |
| absent | 50 (81%) | 9 (75%) |  |  |
| not available | 12 | 0 |  |  |
| **gain/amp(1q)** | 19 (30%) | 5 (42%) | 0.5 |  |
| absent | 44 (70%) | 7 (58%) |  |  |
| not available | 11 | 0 |  |  |
| **del(1p)** | 5 (8.2%) | 2 (17%) | 0.3 |  |
| absent | 56 (92%) | 10 (83%) |  |  |
| not available | 13 | 0 |  |  |
| **bone marrow infiltration at first diagnosis of MM (%)^1^** | 50.0 (30.0–80.0) [0.0–100.0] | 80.0 (80.0–90.0) [70.0–100.0] | 0.002 |  |
| not available | 12 | 3 |  |  |
| **bone marrow infiltration at occurrence of EMD (%)^1^** | 35.0 (7.0–80.0) [0.0–100.0] | 35.0 (1.0–100.0) [1.0–100.0] | >0.9 |  |
| not available | 32 | 5 |  |  |

**Baseline characteristics of patients in the study cohort stratified by patients with EMD and EMD with concurrent PCL**

Values are presented as median (interquartile range) [range] or as n (%), unless otherwise indicated.¹ Percentages calculated among patients with available data.² Percentages may exceed 100% as patients could have multiple sites of organ involvement.³ High-risk cytogenetics defined as del(17p), t(4;14), and t(14;16) according to R-ISS criteria.⁴ Wilcoxon rank sum test; Fisher's exact test; NA; Pearson's Chi-squared test
