# Supplementary figures and images for: Characteristics, treatment regimens, and outcomes of patients with true extramedullary multiple myeloma: a real-world monocentric analysis
Source: Ann Hematol. 2026 Jun 16;105(7):300. doi: 10.1007/s00277-026-07118-6 (PMC13272244; doi:10.1007/s00277-026-07118-6)

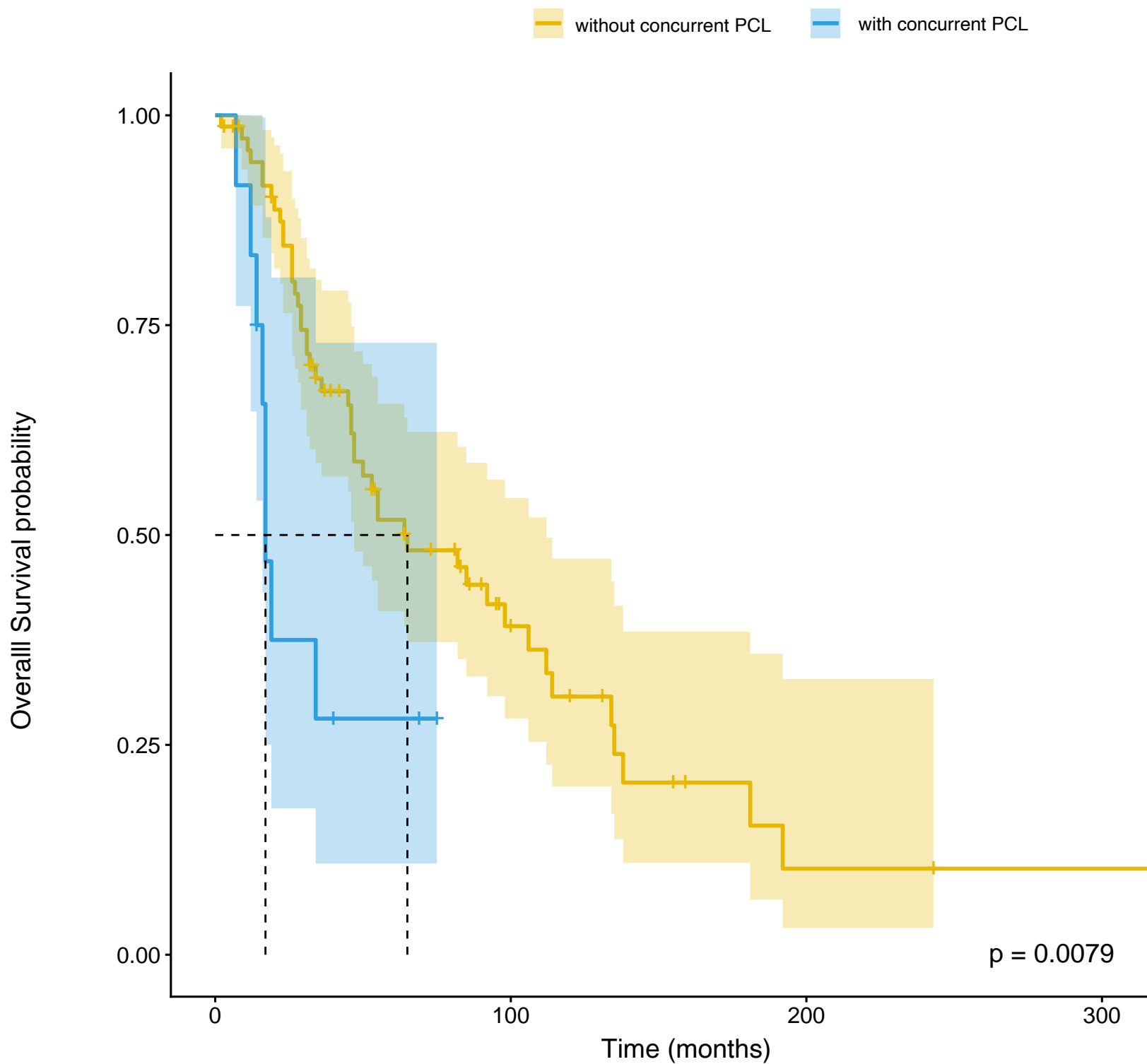

Number at risk

|                            |    |    |   |   |
|----------------------------|----|----|---|---|
| EMD without concurrent PCL | 74 | 15 | 2 | 1 |
| EMD with concurrent PCL    | 12 | 0  | 0 | 0 |

Supplement: Supplementary file 3 — Supplementary file3 Overall survival from EMD diagnosis by concurrent plasma cell leukemia.Kaplan–Meier curves illustrating overall survival from EMD (OS-EMD) in patients with EMD without concurrent PCL (n = 74) and with concurrent PCL (n = 12). OS-EMD differed significantly between the groups (p = 0.002). (PDF 28 KB) [file 277_2026_7118_MOESM3_ESM.pdf]

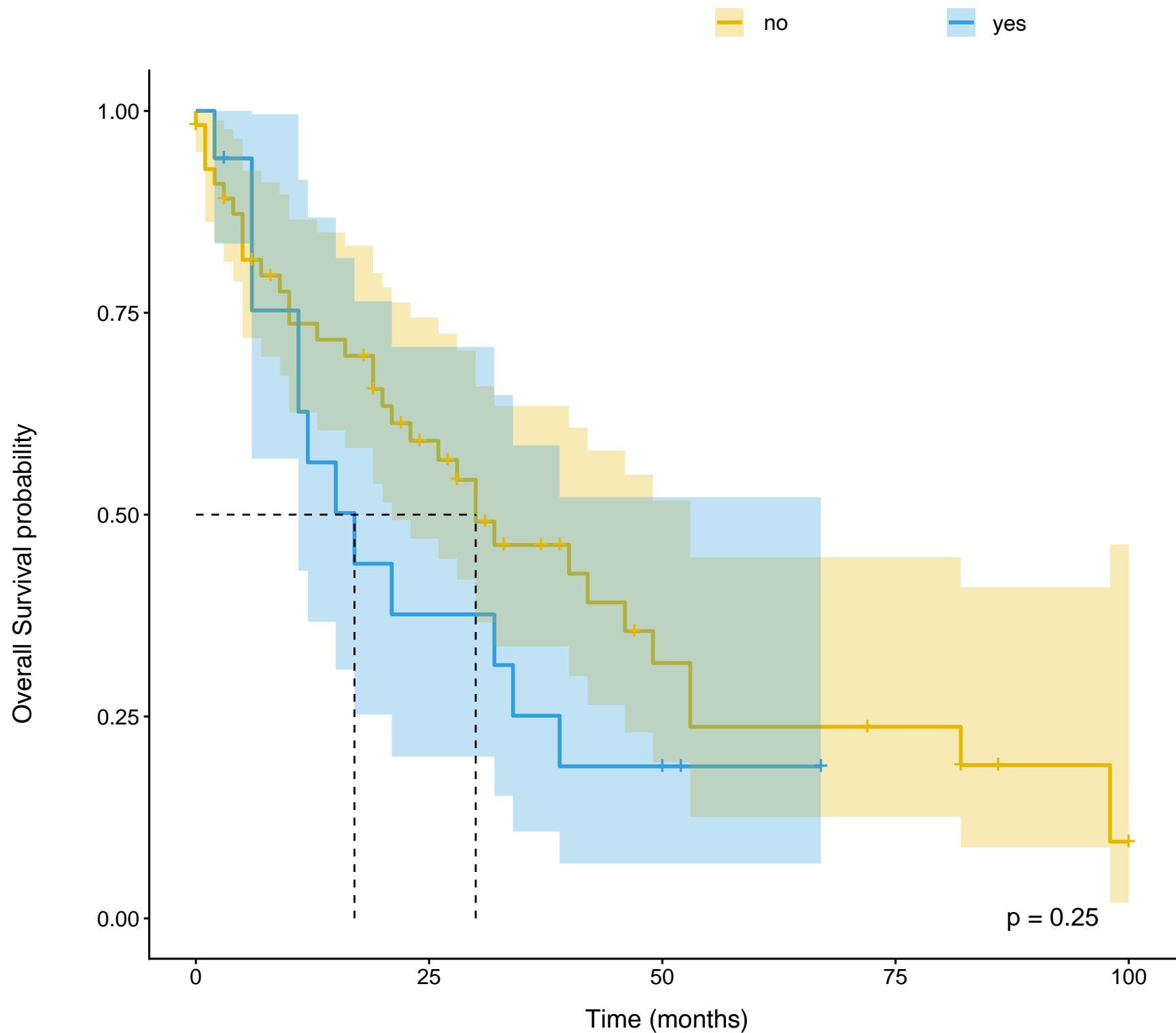

Number at risk

no retroperitoneal  
involvement

57

25

8

5

1

retroperitoneal  
involvement

17

6

3

0

0

Supplement: Supplementary file 4 — Supplementary file4 Survival outcomes according to anatomical sites of EMD.Kaplan–Meier curves illustrating overall survival from the time of EMD diagnosis stratified by anatomical site (A–G): A, retroperitoneal; B, CNS; C, pulmonary; D, cutaneous; E, hepatic; F, muscular; G, lymph node involvement. (PDF 39 KB) [file 277_2026_7118_MOESM4_ESM.pdf]

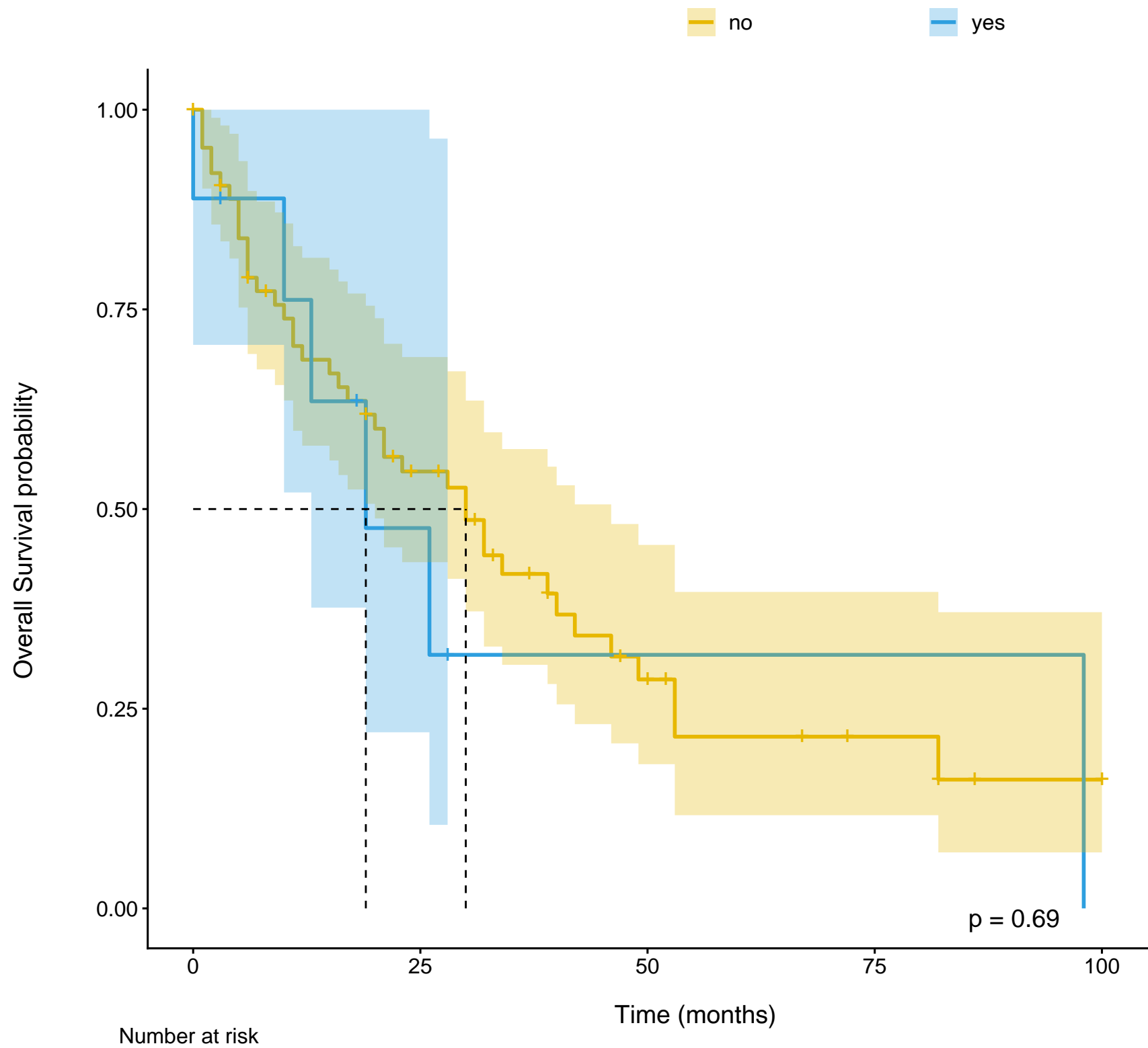

no CNS involvement

65

28

10

4

1

CNS involvement

9

3

1

1

0

Supplement: Supplementary file 5 — Supplementary file5 (PDF 78 KB) [file 277_2026_7118_MOESM5_ESM.pdf]

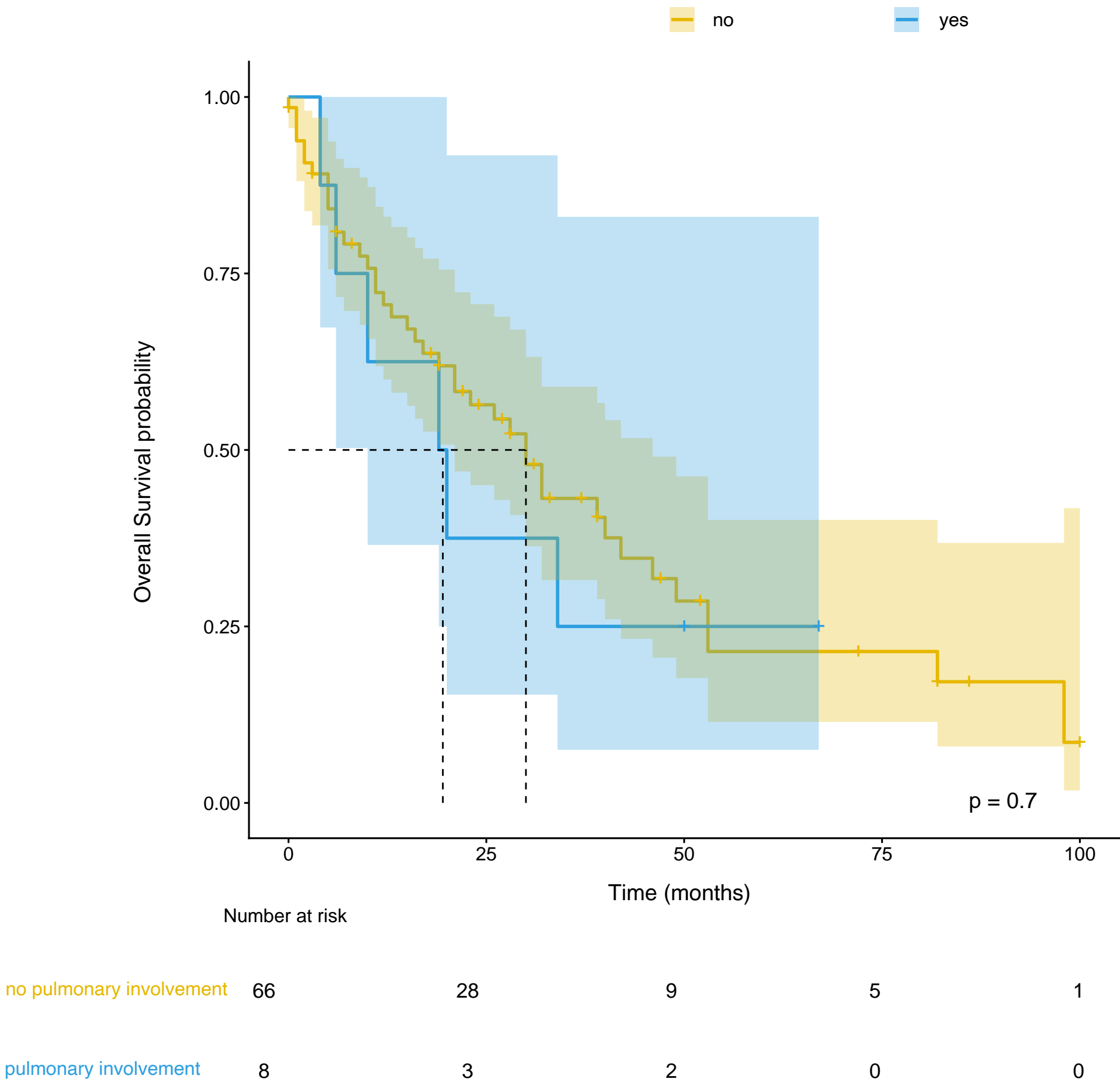

Supplement: Supplementary file 6 — Supplementary file6 (PDF 96 KB) [file 277_2026_7118_MOESM6_ESM.pdf]

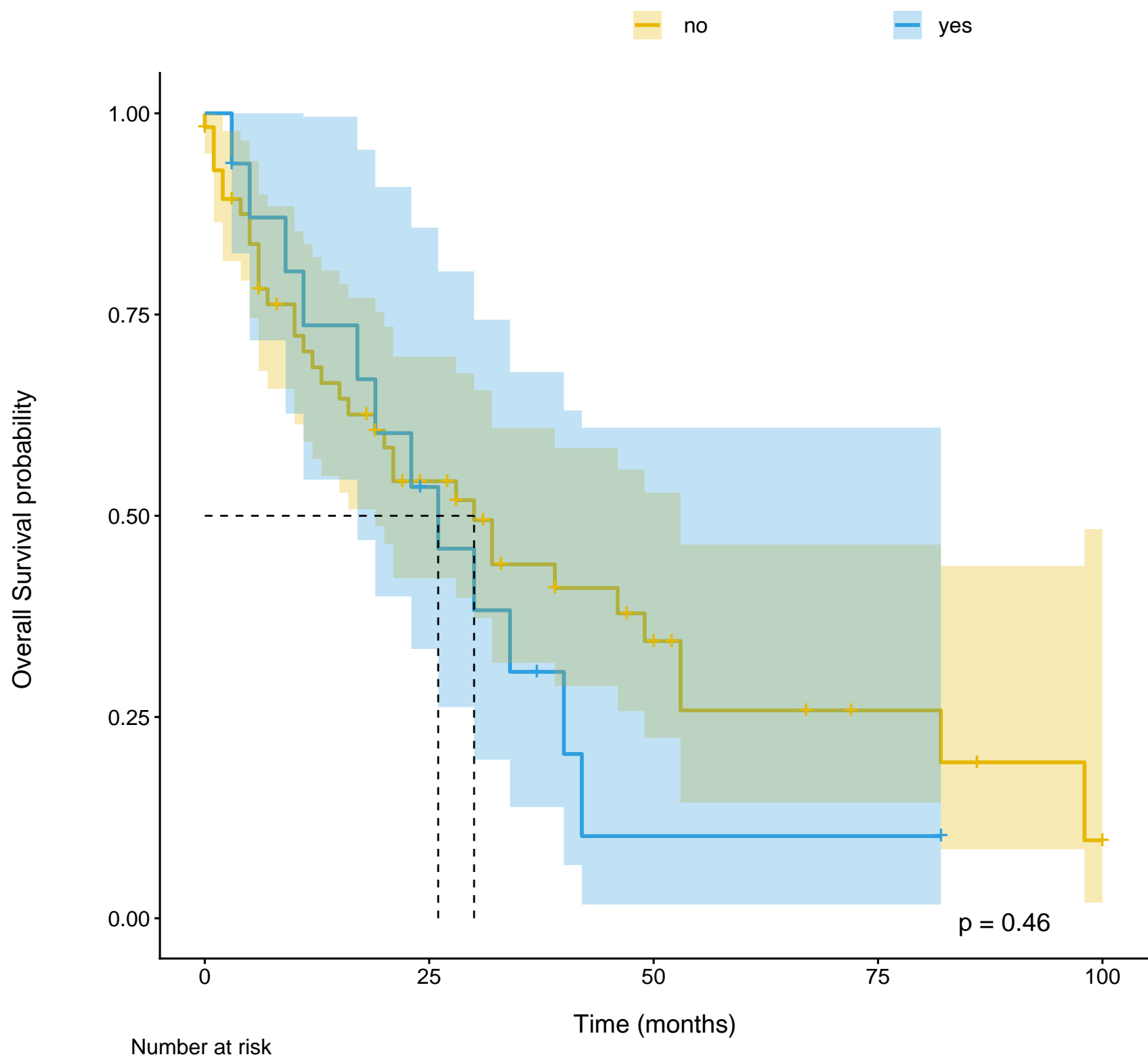

no cutaneous involvement

58

24

10

4

1

cutaneous involvement

16

7

1

1

0

Supplement: Supplementary file 7 — Supplementary file7 (PDF 78 KB) [file 277_2026_7118_MOESM7_ESM.pdf]

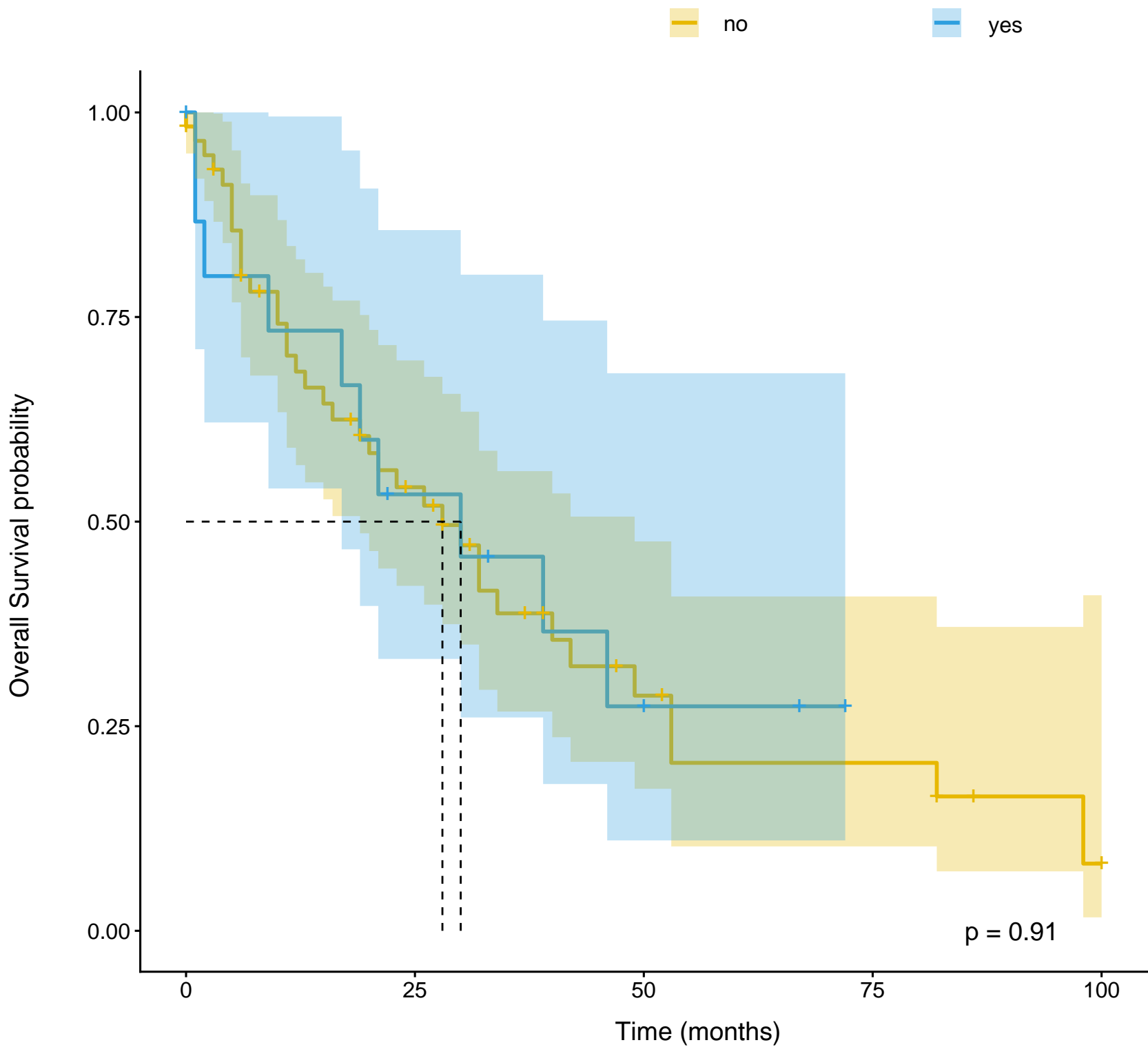

no hepatic involvement

58

24

8

5

1

hepatic involvement

16

7

3

0

0

Supplement: Supplementary file 8 — Supplementary file8 (PDF 78 KB) [file 277_2026_7118_MOESM8_ESM.pdf]

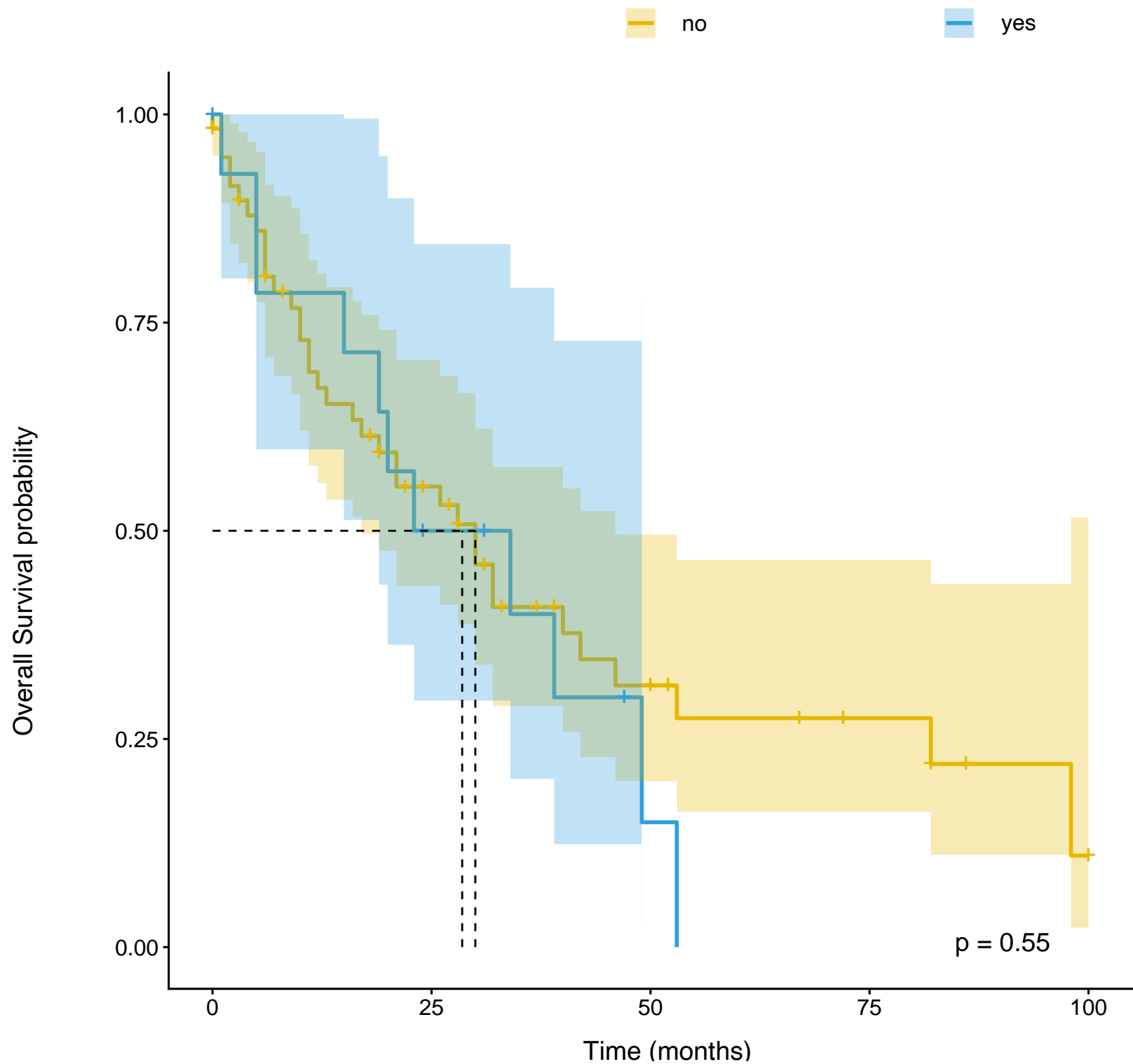

Number at risk

no muscular involvement

59      25      10      5      1

muscular involvement

15      6      1      0      0

Supplement: Supplementary file 9 — Supplementary file9 (PDF 26 KB) [file 277_2026_7118_MOESM9_ESM.pdf]

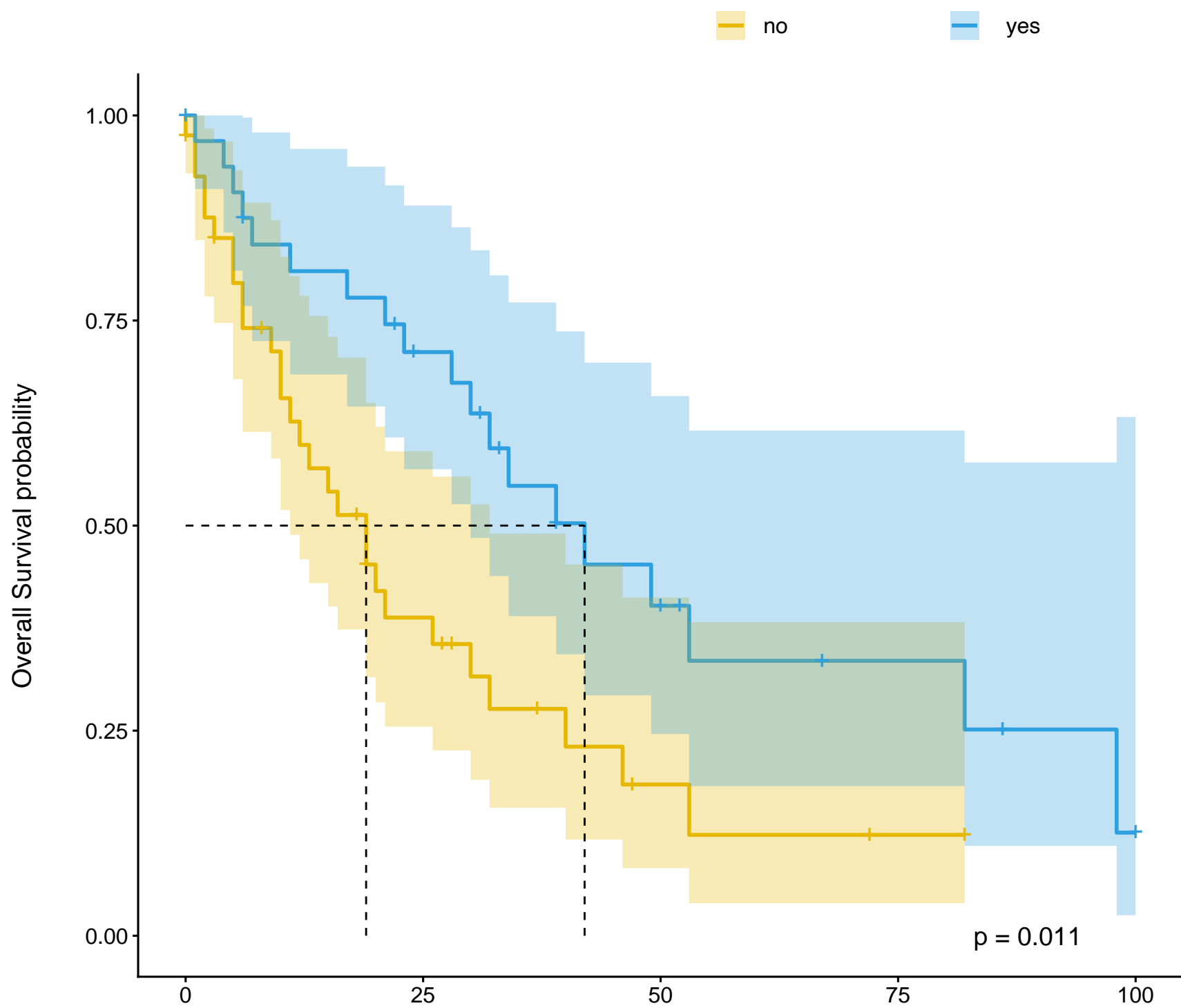

| Number at risk            |    |    |   |   |   |
|---------------------------|----|----|---|---|---|
| no lymph-node involvement | 41 | 12 | 3 | 1 | 0 |
|                           |    |    |   |   |   |
| lymph-node involvement    | 33 | 19 | 8 | 4 | 1 |
|                           |    |    |   |   |   |

Supplement: Supplementary file 10 — Supplementary file10 (PDF 60 KB) [file 277_2026_7118_MOESM10_ESM.pdf]

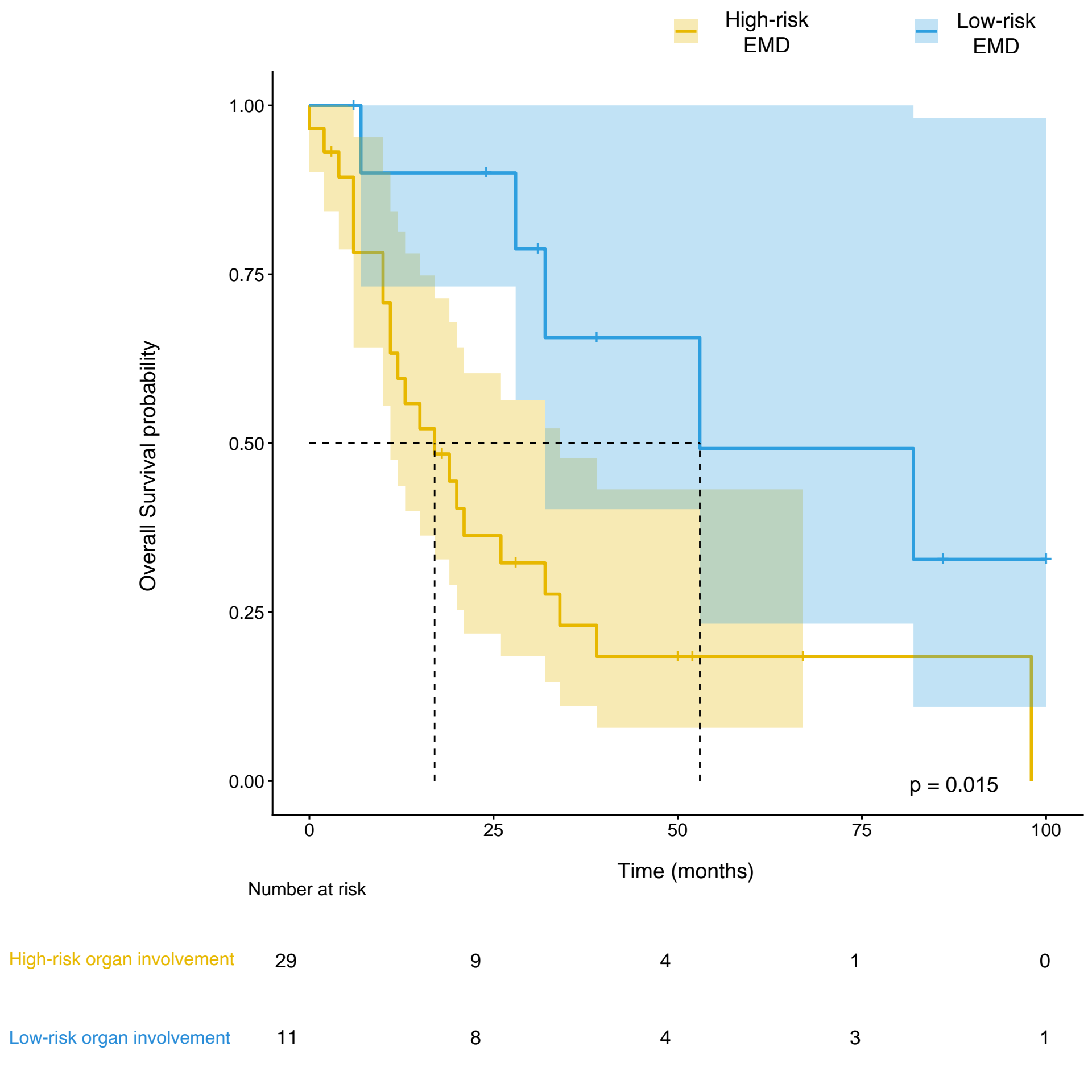

Supplement: Supplementary file 11 — Supplementary file11 Overall survival from the time of EMD diagnosis according to anatomical high-risk and low-risk organ involvement. Kaplan–Meier curves comparing overall survival from EMD diagnosis (OS-EMD) between patients with high-risk EMD (HR; CNS, pulmonary, or retroperitoneal involvement; n = 29) and those with low-risk EMD (LR; lymph node involvement only; n = 11). Median OS-EMD was significantly shorter in the HR group compared with the LR group (17 vs. 53 months; p = 0.015). (PDF 32 KB) [file 277_2026_7118_MOESM11_ESM.pdf]

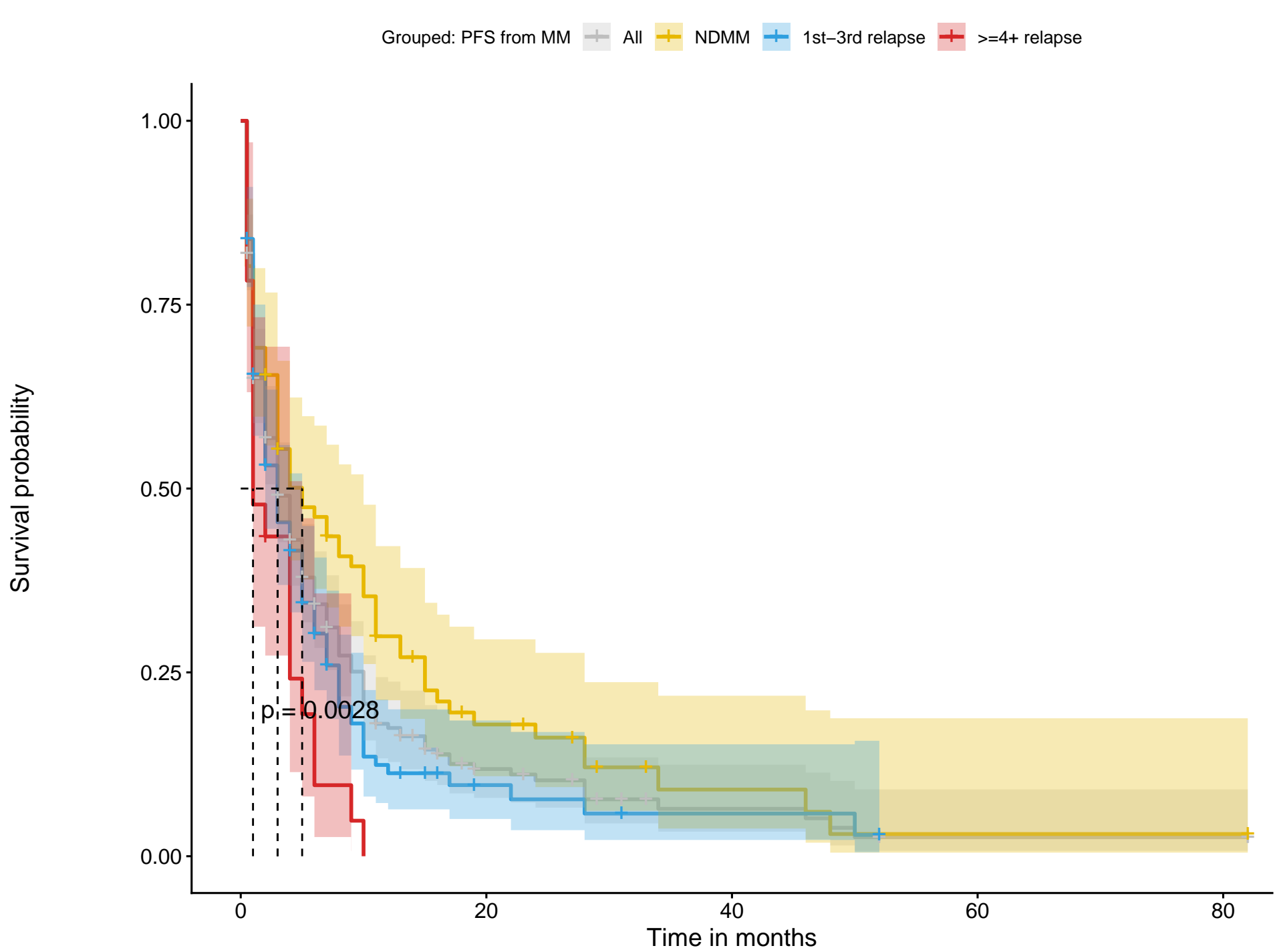

Number at risk

|                 |     |    |   |   |   |
|-----------------|-----|----|---|---|---|
| All             | 216 | 16 | 5 | 1 | 1 |
| NDMM            | 81  | 11 | 3 | 1 | 1 |
| 1st-3rd relapse | 112 | 5  | 2 | 0 | 0 |
| ≥4th relapse    | 23  | 0  | 0 | 0 | 0 |

Supplement: Supplementary file 12 — Supplementary file12 Progression-free survival by line of therapy from EMD diagnosis.Kaplan–Meier curves illustrating progression-free survival (PFS) across treatment lines initiated from the time of EMD occurrence onwards (n=216 treatment lines in 82 patients). Groups are shown for newly diagnosed MM (NDMM), 1st-3rd relapse, and ≥4th relapse settings. Median PFS declined progressively with increasing lines of therapy (p=0.0028) (PDF 39 KB) [file 277_2026_7118_MOESM12_ESM.pdf]
